# Reversible homodimerization of a small leucine-rich proteoglycan PRELP modulates the interactions with TGFβ1 and IGFI-R

**DOI:** 10.1016/j.jbc.2026.113523

**Published:** 2026-09-08

**Authors:** Hirofumi Kosuge, Makoto Nakakido, Daisuke Kuroda, Kouhei Tsumoto

**Affiliations:** 1 Department of Bioengineering, School of Engineering, The University of Tokyo, Bunkyo-ku, Tokyo, Japan; 2 Department of Chemistry and Biotechnology, School of Engineering, The University of Tokyo, Bunkyo-ku, Tokyo, Japan; 3 Department of Biosciences, College of Humanities and Sciences, Nihon University, Setagaya-ku, Tokyo, Japan; 4 The Institute of Medical Science, The University of Tokyo, Minato-ku, Tokyo, Japan

**Keywords:** dimerization, mass photometry, MD Simulation, PRELP, small leucine-rich repeat proteoglycans, Protein Design, Protein-Protein Interaction, SEC-MALS, spatial aggregation propensity, surface plasmon resonance

## Abstract

Small leucine-rich proteoglycans (SLRPs) are responsible for various biological functions *via* their multiple interactions with various proteins. Although some SLRPs form homodimers, it remains unclear how homodimerization affects the molecular functions of SLRPs. Herein, we report the molecular basis and functional significance of homodimerization of proline/arginine-rich end leucine-rich repeat protein (PRELP), which is a member of SLRP family. We used biophysical techniques to validate the premise that PRELP forms a reversible homodimer in a concentration-dependent manner. Because the spatial aggregation propensity calculation indicated that the concave surface of the leucine-rich repeat domain of PRELP is highly hydrophobic, we speculated that this hydrophobicity predominantly drives PRELP homodimerization. To address this hypothesis, we replaced several amino acid residues that showed high spatial aggregation propensity values with charged residues identified by the Rosetta energy calculation, which resulted in disruption of PRELP homodimerization. Molecular dynamics simulation further verified the molecular basis of the monomerizing mutation as well as an unexpected dimerizing mutation. To demonstrate the influence of reversible homodimerization on PRELP interactions, we conducted interaction analysis using the designed PRELP mutants. We found that the monomerizing mutation of PRELP enhanced interactions with transforming growth factor β1 and insulin-like growth factor I receptor, suggesting that the functions of PRELP that rely on these interactions are moderated *via* reversible homodimerization.

The small leucine-rich proteoglycan (SLRP) family consists of 18 secreted proteins that contain the leucine-rich repeat (LRR) structure and are responsible for various biological phenomena (1, 2, 3). Among these proteins, decorin and biglycan are thought to form reversible homodimers through the LRR core domain (4, 5, 6), while osteomodulin, fibromodulin, and chondroadherin exist as monomers and likely do not form dimers stably in solution (7, 8). The crystal structures of decorin and biglycan show that the N-terminal parts of the concave surface of the LRR domain form the dimer interface (4, 5). Although it remains unclear whether SLRP proteins function as monomers or dimers, results of previous studies suggest that decorin interacts as a monomer with collagen (6, 9, 10) and low-density lipoprotein receptor-related protein 1 (11) *via* the LRR concave surface. In a mutation-based study, Tashima *et al.* found that a monomeric osteomodulin bound to collagen through electrostatic interactions *via* the LRR concave surface and regulated the fibril formation of collagen (8). These observations suggest that homodimerization of SLRPs affects ligand binding and molecular functions. Therefore, it is important to understand the molecular basis by which the oligomeric state of SLRPs governs biological functions.

Proline/arginine-rich end leucine-rich repeat protein (PRELP) is a member of class II SLRP family, and it has diverse molecular functions, including tumor suppressive roles (12, 13, 14, 15, 16, 17). We previously reported that the functions of PRELP rely on its weak affinity interactions with multiple proteins *via* the LRR domain (18, 19). PRELP interacts with transforming growth factor β1 (TGFβ1) and insulin-like growth factor I receptor (IGFI-R), which are involved in cancer progression, likely *via* the N-terminal part of the LRR domain (18). The interaction between PRELP and TGFβ1 leads to inhibition of TGFβ1 signaling (16, 20). However, whether PRELP exists in monomeric or dimeric states and whether the oligomeric states influence the target binding properties and molecular functions of PRELP remain unclear. Therefore, studies of the roles of PRELP oligomerization are crucial to understanding the complex biological phenomena triggered by the PRELP interaction network.

Herein, we investigated the monomer and homodimer status of PRELP in solution using biophysical methods, including size-exclusion chromatography–multi-angle light scattering (SEC-MALS) and mass photometry. We also combined computational analysis based on spatial aggregation propensity (SAP) calculation and molecular dynamics (MD) simulations with experimental characterization *via* structure-based design of monomeric and dimeric PRELP mutants to obtain mechanistic insights about the reversible homodimerization of PRELP. Using the designed PRELP mutants, we then assessed how PRELP homodimerization influences its interactions with target proteins and its tumor suppressive effects in order to identify a molecular mechanism that explains the functional role of the monomer-dimer equilibrium of PRELP.

## Results

### Biophysical characterization of concentration-dependent reversible homodimerization of PRELP

To determine whether PRELP exists as monomers or dimers in solution, we prepared the recombinant protein and evaluated the oligomeric states of PRELP using biophysical analysis. The SEC profile during the purification of PRELP revealed two partially overlapping peaks at around 150 to 200 ml elution volume (Fig. 1*A*); both peaks contained PRELP proteins with almost identical molecular weights in the denatured condition, as shown by Coomassie staining of sodium dodecyl sulfate-polyacrylamide gel electrophoresis gels (Fig. 1*B*) and Western blot analysis (Fig. 1*C*). We considered these two peaks to be the monomeric and dimeric states. Analytical SEC analysis showed that the ratio of the two peaks changed in a concentration-dependent manner: lower concentrations contained a larger proportion of the putatively monomeric peak 2 relative to the putatively dimeric peak 1 (Fig. 1*D*). The SEC-MALS analysis of PRELP in solution showed that the molar mass increased from monomer (theoretically around 50 kDa) to homodimer in a concentration-dependent manner (Fig. 1*E*), suggesting the concentration-dependent equilibrium of PRELP between monomeric and dimeric states as expected.

**Figure 1:**
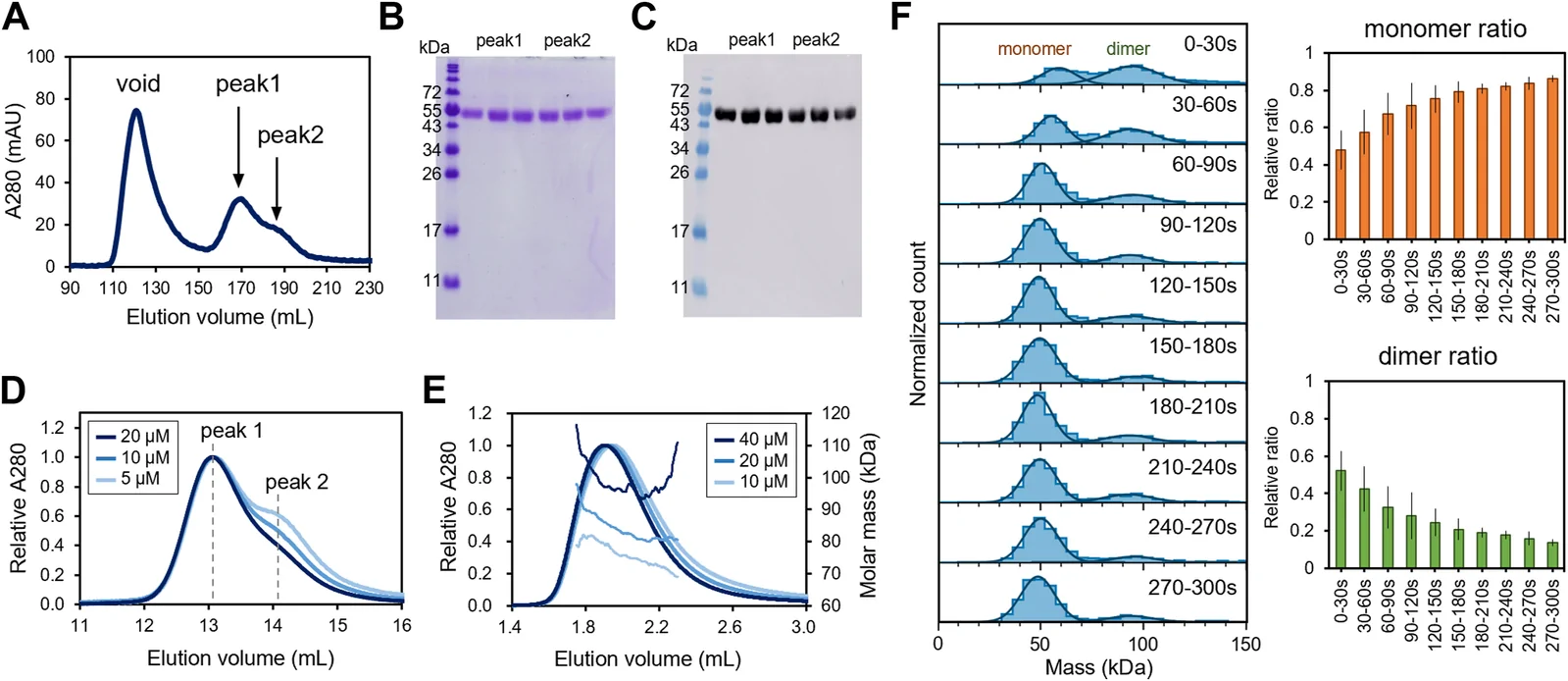
**Characterization of concentration-dependency and reversibility of PRELP homodimerization.***A*, SEC purification profile of recombinant PRELP protein. *B*, coomassie staining of sulfate-polyacrylamide gel electrophoresis gels of the purified PRELP. C, Western blot of the purified PRELP. The proteins were detected with anti-DDDDK-tag mAb HRP-DirecT. *D*, analytical SEC. 5, 10, and 20 μM of PRELP proteins were injected. Line darkness indicates concentration, with the darkest line indicating the highest concentration. *E*, size-exclusion chromatography–multi-angle light scattering. 10, 20, and 40 μM of PRELP proteins were injected. Line darkness indicates concentration, with the darkest line indicating the highest concentration. *F*, mass photometry analysis. Mass distribution was measured in a time-dependent manner immediately after 10-fold dilution of 500 nM of PRELP proteins into buffer. The average values ± standard deviations of the relative abundance ratio of monomers and dimers in four technical replicates are shown. PRELP, proline/arginine-rich end leucine-rich repeat protein.

We then assessed the reversibility of PRELP homodimerization using mass photometry, which measures the mass of single protein molecules in solution based on light scattering (21). The molar mass and abundance ratio of monomers and dimers were consecutively monitored immediately after 10-fold dilution of 500 nM of PRELP samples with buffer. In the consecutive recordings, a time-dependent decrease of the dimer population was observed (Fig. 1*F*). This gradual decrease in the dimer ratio showed that the monomer–dimer equilibrium shifted toward the monomer caused by the reduced protein concentration upon dilution, demonstrating that the PRELP homodimer is reversibly dissociated into monomers in the concentration-dependent manner.

### Contribution of the hydrophobic interface to PRELP homodimerization

Because the importance of hydrophobic residues for promoting protein-protein interactions is generally accepted (22, 23, 24, 25), to assess the driving force of PRELP homodimerization from the perspective of hydrophobicity we conducted analytical SEC in the presence of three detergents, n-dodecyl-β-D-maltoside (DDM), Tween-20, and 3-((3-cholamidopropyl)dimethylammonio)-1-propanesulfonate (CHAPS) and 8-anilino-1-naphthalenesulfonic acid (ANS), which is a fluorescent probe that binds to exposed hydrophobic surfaces of proteins. Addition of 0.05% (v/v) Tween-20, 0.01% (w/v) DDM, 0.1% (w/v) CHAPS, or 80 μM ANS to the PRELP sample promoted monomerization (Fig. 2*A*), probably because these additives cover and stabilize exposed hydrophobic surface. Although CD measurements showed that the secondary structure of PRELP was not significantly changed in the presence of Tween-20 and CHAPS, it should be noted that DDM and ANS slightly affected the spectra (Fig. S1). To assess the contribution of electrostatic interactions to PRELP homodimerization, analytical SEC was also performed at different salt concentrations, and no substantial changes in the monomer-dimer ratio were observed (Fig. S2). These results suggest that a dominant driving force of PRELP homodimerization is hydrophobicity.

**Figure 2:**
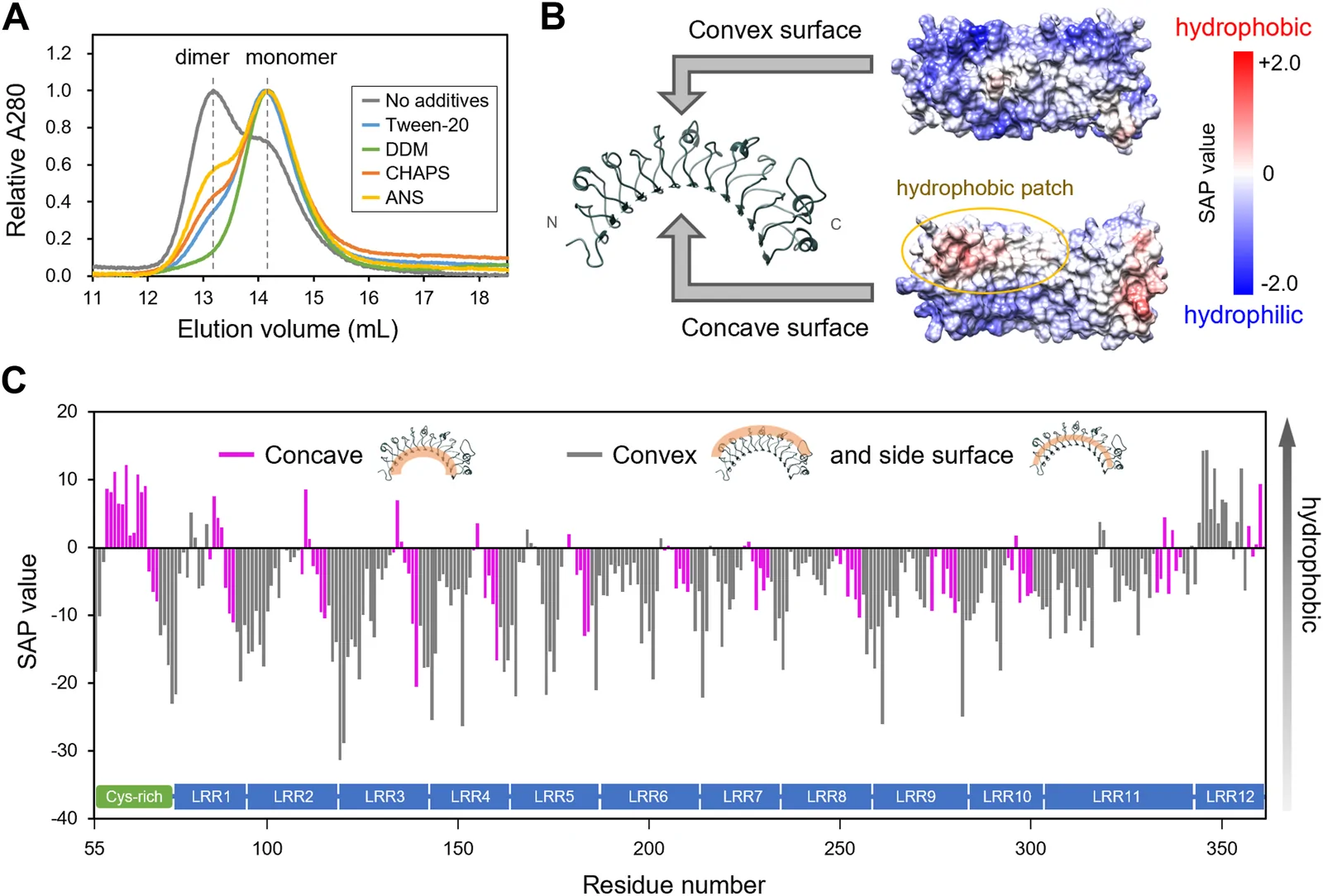
**Evaluation of surface hydrophobicity of PRELP and its contribution to dimerization.***A*, Analytical SEC. 4 μM of PRELP samples containing 0.05% (v/v) Tween-20 (*blue*), 0.01% (w/v) dodecyl-β-D-maltoside (*green*), 0.1% (w/v) CHAPS (*orange*), 80 μM ANS (*yellow*), or not (*gray*) were loaded. *B*, SAP calculation of the PRELP monomer. The calculated SAP value of each atom was mapped onto a model structure of the PRELP monomer in the SWISS-MODEL repository. A hydrophobic patch on the concave surface at the N-terminus is indicated by a *yellow circle*. *C*, SAP value of each amino acid residue of the PRELP monomer; the residues on the concave surface are colored in *magenta*. The side surface refers to the edge of the horseshoe-shaped LRR domain located between the convex and concave face. The residue numbering is based on the mature human PRELP protein sequence, with the N-terminal residue after signal peptide cleavage designated as residue 1. LRR, leucine-rich repeat; PRELP, proline/arginine-rich end leucine-rich repeat protein; SAP, spatial aggregation propensity.

To identify hydrophobic regions on the PRELP surface, we calculated the SAP (26), which characterizes the extent of aggregation-prone hydrophobic patches exposed on the protein surface. The SAP was analyzed using the model structure of the PRELP monomer in the SWISS-MODEL repository (27) as well as the crystal structures of decorin and biglycan for comparison. The calculated SAP value of each atom was mapped onto the monomer structures of PRELP (Fig. 2*B*). The SAP value of each residue in PRELP was also calculated by summing the SAP values for all component atoms (Fig. 2*C*). The analysis showed high hydrophobicity of the concave surface of the LRR domain in the PRELP monomer, and it identified a large hydrophobic patch at the N-terminus compared with decorin and biglycan (Fig. S3). To assess whether the results of SAP calculation were affected by uncertainty of the used model structure, the SAP was also analyzed using a PRELP structure predicted by AlphaFold3 (28), and the overall trend of SAP profile remained unchanged (Fig. S4). Given that the crystal structures of decorin and biglycan indicate that the N-terminal part of the concave surface of the LRR domain forms the dimer interface (4, 5), we expected that PRELP also forms a homodimer *via* the N-terminal part of the LRR concave surface and that the hydrophobic patch at the N-terminus of the concave surface identified by the SAP calculation may contribute to the homodimerization of PRELP.

### Computational design of monomeric mutants of PRELP

Because controlling the oligomeric states of PRELP would be helpful for investigating the mechanism and functional significance of the homodimerization of PRELP, we designed monomeric mutants of PRELP. We hypothesized that introduction of hydrophilic mutations to the hydrophobic patch at the N-terminal concave surface of the LRR domain would disrupt the homodimerization of PRELP. Amino acid residues with positive SAP values excluding the C-terminus (within amino acid residues 55–300) are listed in Table S1 as relatively hydrophobic residues. Among them, all amino acid residues with SAP values > 2 in the concave surface (except for cysteine and charged amino acids) were computationally mutated. Using the Rosetta energy calculation (29), we predicted the influence of a single substitution of a charged amino acid residue (Lys (K), Arg (R), Asp (D), or Glu (E)) for these selected residues on the structural stability of the PRELP monomer and then identified single mutations at six positions (Tyr58 (Y58), Pro60 (P60), Phe63 (F63), Ser65 (S65), Ala66 (A66), Gln155 (Q155)) with negative ΔΔG values (Table 1). It should be noted that the structural stability of the PRELP dimer was not considered in this energy calculation. With the exception of the Q155 residue, the designed residues clustered at the N-terminus of the concave surface of the LRR domain (Fig. 3*A*). Based on a homology modelling structure of the human PRELP homodimer constructed using MODELLER (30) (Fig. 3*B*), these six amino acid residues likely contribute to the formation of the hydrophobic dimer interface of PRELP (Fig. 3*C*). These results suggest that substitution of these amino acid residues with charged hydrophilic residues would disrupt the homodimerization of PRELP.

**Table 1:** Single mutations with negative ΔΔG values calculated using Rosetta software

Amino acid residue in WT^a^	SAP value in WT^b^	Mutation	ΔΔG _MUT-WT_ (REU)^c^
Tyr58	8.61	Asp	−0.465
Pro60	11.10	Asp	−0.090
Phe63	12.14	Glu	−0.245
Ser65	2.16	Arg	−5.594
		Glu	−4.984
		Lys	−3.737
		Asp	−1.737
Ala66	10.76	Asp	−1.460
		Glu	−1.265
		Arg	−0.305
Gln155	3.53	Glu	−3.265
		Arg	−1.924

a The residue numbering is based on the mature human PRELP protein sequence, with the N-terminal residue after signal peptide cleavage designated as residue 1.

b The SAP value for each amino acid residue was calculated by summing the SAP values for all component atoms.

c The energy calculation was carried out using a homology modelling structure of the human PRELP monomer in the SWISS-MODEL repository.

**Figure 3:**
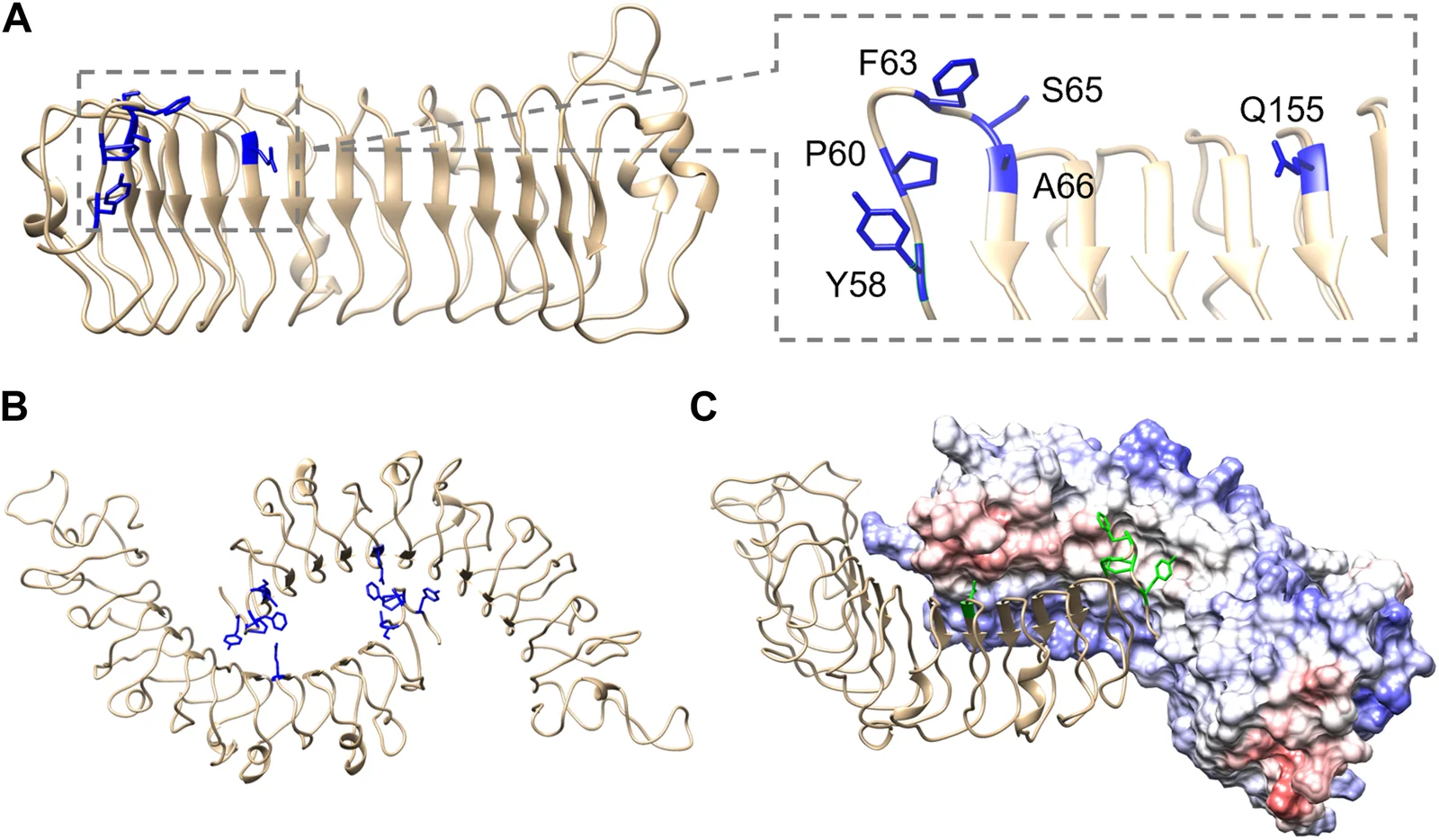
**Identification of mutation sites based on Rosetta energy calculation.***A*, amino acid residues that showed negative ΔΔG values upon single mutation by charged residues. The residues are shown in *blue* on a model PRELP WT monomer structure. The residue numbering is based on the mature human PRELP protein sequence, with the N-terminal residue after signal peptide cleavage designated as residue 1. *B*, PRELP WT homodimer structure modelled by MODELLER. *C*, A model of PRELP WT dimer structure superposed on the SAP-mapped structure. The identified mutation sites are shown in *green*. PRELP, proline/arginine-rich end leucine-rich repeat protein.

### Experimental characterization of homodimerization of the designed PRELP mutants

Based on the results of Rosetta energy calculation shown in Table 1, PRELP Mut5 and Mut6, which contain five mutations (Y58D (*i.e.*, Y58 to D58), P60D, F63E, S65E, and A66D) and six mutations (Y58D, P60D, F63E, S65E, A66D, and Q155E), respectively, were produced as recombinant proteins. PRELP Mut5 exhibited a larger proportion of the monomer peak in the SEC purification profile compared with PRELP WT (Fig. 4*A*), suggesting that this mutation successfully promotes the monomerization of PRELP. In contrast, PRELP Mut6 unexpectedly did not show a high monomer ratio (Fig. S5*A*). CD measurements showed that these mutations did not largely affect the secondary structure of PRELP (Fig. S5*B*).

**Figure 4:**
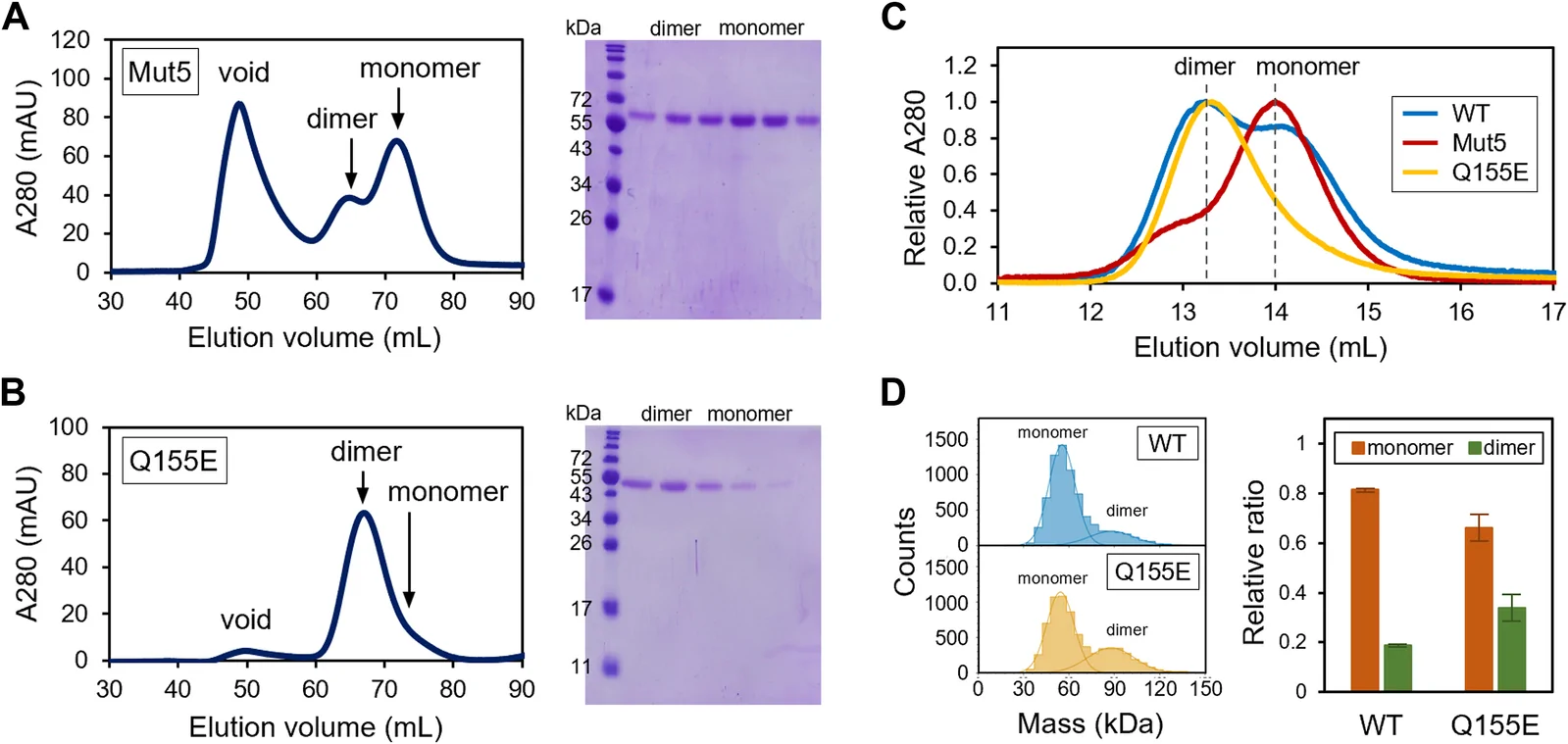
**Purification and *in vitro* characterization of PRELP mutants.***A* and *B*, SEC purification profile and Coomassie staining of sulfate-polyacrylamide gel electrophoresis gels of (*A*) PRELP Mut5 (Y58D, P60D, F63E, S65E, and A66D) and (*B*) PRELP Q155E. *C*, analytical SEC. 5 μM of PRELP WT (*blue*), Mut5 (*red*), and Q155E (*yellow*) were loaded. *D*, mass photometry analysis of PRELP WT and Q155E. Mass distribution was measured without dilution after the samples were adjusted to 50 nM and incubated for 1 h to allow the monomer-dimer equilibrium to be reached. The average values ± standard deviations of the relative abundance ratio of monomers and dimers in three (WT) or five (Q155E) technical replicates are shown. PRELP, proline/arginine-rich end leucine-rich repeat protein.

To validate the contribution of each single mutation to the monomer-dimer equilibrium, six single mutants of PRELP (Y58D, P60D, F63E, S65E, A66D, and Q155E) were also prepared (Figs. 4*B* and S6). Based on CD measurements, these mutations did not lead to large differences in the secondary structure of PRELP (Fig. S7). Although the SEC purification profile indicated that Y58D had a higher monomer ratio than the other single mutants, the monomer ratio of any single mutant was not comparable to that of Mut5. This result suggests that the cluster of negatively charged amino acids in proximity is important for the monomerizing effects of the Mut5 mutation. As expected from the purification profile of Mut6, which did not successfully achieve monomerization, Q155E showed a quite high dimer ratio (Fig. 4*B*). It should be also noted that the peak heights of the void fraction in the SEC profile seemed to correlate with the monomer ratio of each mutant, indicating that exposure of the hydrophobic concave surface likely causes the formation of high-molecular-weight species.

We subsequently obtained Mut5, a mutant with a high monomer ratio, and Q155E, a mutant with a high dimer ratio, and conducted analytical SEC using the same concentration (5 μM) of purified recombinant PRELP WT, Mut5, and Q155E. The results confirmed that the monomer ratio increased in the order Q155E < WT < Mut5 (Fig. 4*C*). We also compared the monomer-dimer equilibrium of PRELP WT, Mut5, and Q155E using mass photometry. After the samples were adjusted to 50 nM and incubated at room temperature for 1 h to allow the equilibrium to be reached, the abundance ratio of monomers and dimers were measured. We observed no significant differences in monomer-dimer ratio between WT and Mut5 (Figs. 4*D* and S8) since the dimer population was too low even at the maximum recommended concentration for mass photometry (50 nM), which makes it difficult to accurately compare the extent of dimerization. Q155E showed a higher dimer ratio than WT did (Fig. 4*D*), and the dissociation constant (*K*_D_) of homodimerization of PRELP Q155E calculated based on the ratio of monomers and dimers was 52.3 ± 21.3 nM, approximately three times smaller than that of WT (148 ± 8 nM).

### Molecular mechanism of PRELP homodimerization

To explore the molecular mechanism of PRELP homodimerization, we performed triplicate MD simulations, with different initial velocities, using model homodimer structures of PRELP WT, Mut5, and Q155E. We checked the convergence of the simulations by calculating the RMSD values of Cα atoms (Fig. S9). To determine whether PRELP homodimers are stabilized or dissociated into monomers in the simulation, we calculated inter-chain distance between Cα atoms of two residues (position 68 and 157) located in close proximity in the dimer interface of the initial structures (Fig. 5*A*). We did not observe dissociation of the PRELP WT dimer throughout the simulations, which supports the reliability of the model dimer structure, although a temporary increase in the distance between monomers was observed. In contrast, distance and fluctuation between two monomer chains was greater in PRELP Mut5 than in PRELP WT throughout the simulations, which suggests lower stability of the Mut5 homodimerization. We also observed complete dissociation of the Mut5 homodimer in the run-3 simulation (Fig. S10). These results suggest that introduction of charged mutations to the hydrophobic dimer interface destabilizes the dimer structure, leading to partial dimer dissociation followed by stochastic complete dissociation into monomers (Fig. 5*B*).

**Figure 5:**
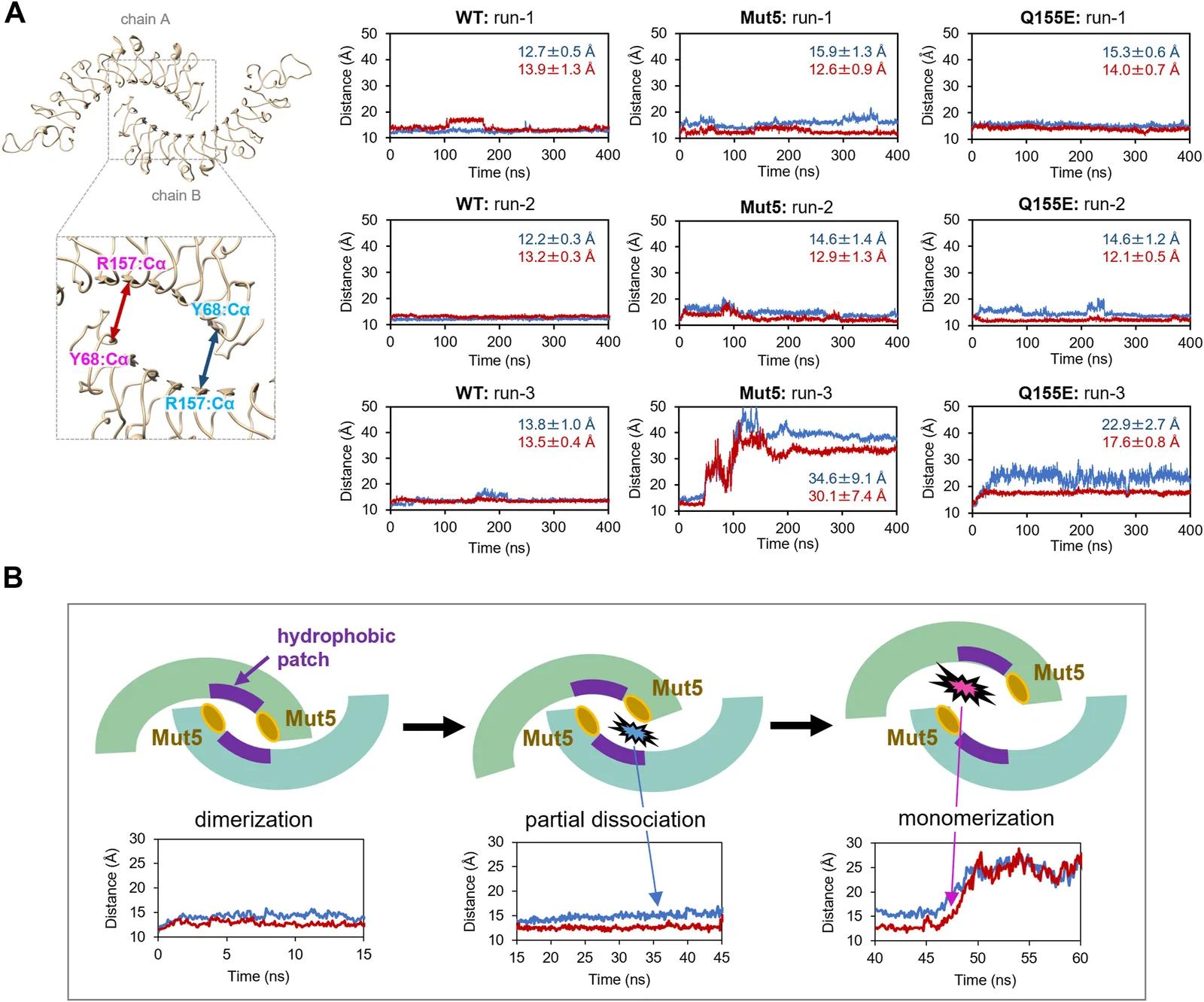
**MD simulation of PRELP homodimers.***A*, distance fluctuations between Cα atoms of Y68 in a monomer chain and R157 in the other monomer chain from simulations. Averaged values of the distance with standard deviation are shown. *B*, proposed mechanism of the dissociation process of PRELP Mut5 (Y58D, P60D, F63E, S65E, and A66D) based on distance fluctuations in the run-3 simulation. PRELP, proline/arginine-rich end leucine-rich repeat protein.

The averaged distance between two monomer chains of PRELP Q155E was relatively greater than that of PRELP WT, although the time-dependent fluctuation of the distance was similar (Fig. 5*A*). Although the effect of Q155E was not as great as that seen in Mut5, the results suggest lower stability of Q155E homodimerization compared with that of WT. This finding is contrary to the experimental result that Q155E mutation enhanced PRELP dimerization (Fig. 4, *C* and *D*). Interestingly, we found that the initial dimer structure of Q155E was partially disrupted and changed into unusual dimeric conformation in the run-3 simulation, but subsequent complete dissociation was not observed throughout the simulation (Fig. S11). After the partial disruption, the E155 residue of one monomer chain was continuously located in close proximity to the R72 residue of the other chain, and side chains of these residues appeared to interact with each other (Fig. 6*A*). We hypothesize that the E155-R72 interaction stabilized the partially dissociated Q155E homodimer, resulting in the high dimer ratio in the analytical SEC and mass photometry experiments.

**Figure 6:**
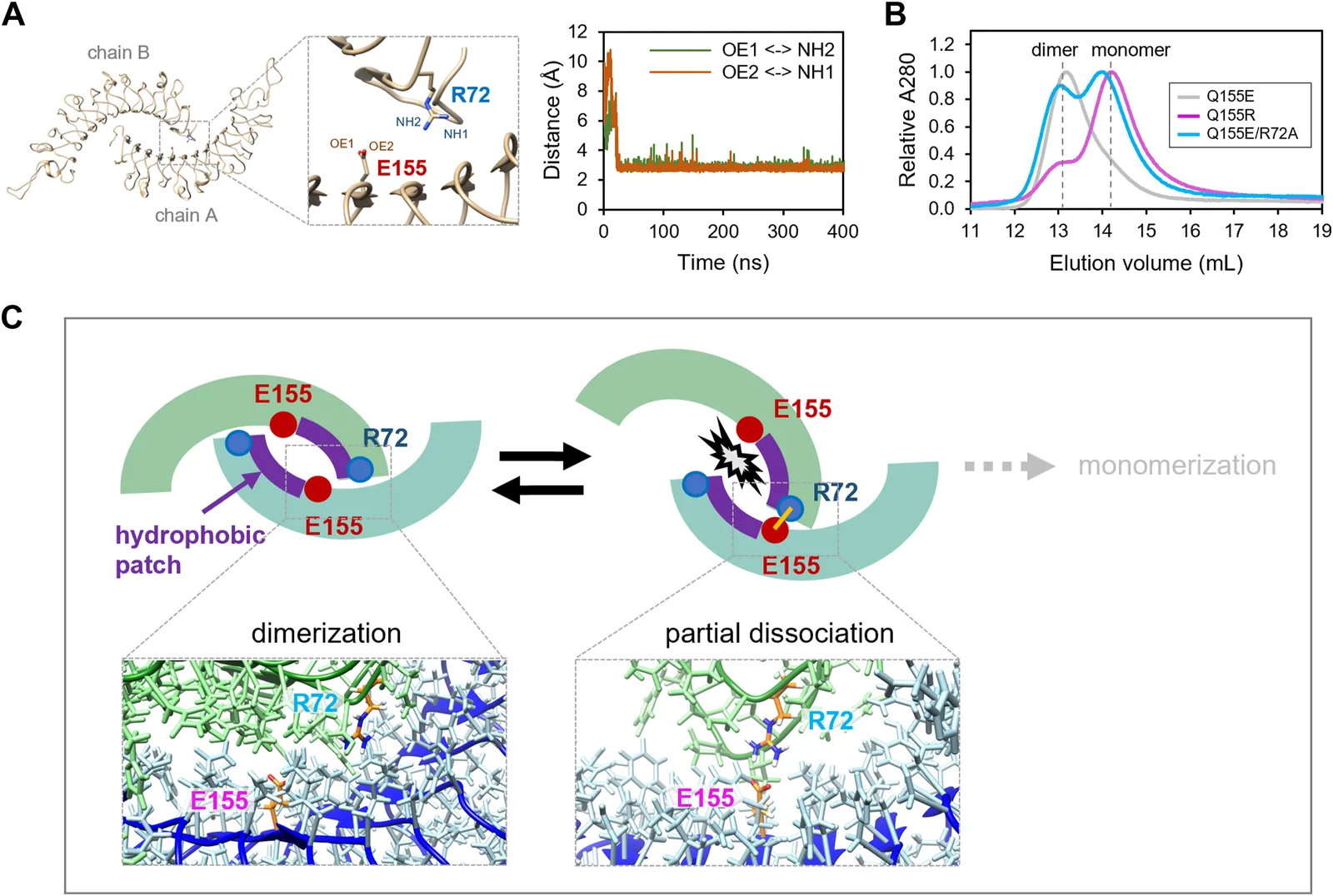
**Investigation of the molecular basis for stable homodimerization of PRELP Q155E.***A*, distance fluctuations between oxygen atoms of the E155 sidechain in a monomer chain and nitrogen atoms of the R72 sidechain in the other monomer chain in the run-3 MD simulation of PRELP Q155E. *B*, Analytical SEC. 5 μM of PRELP Q155E (*gray*), Q155R (*magenta*), and Q155E/R72A (*light blue*) were loaded. *C*, proposed mechanism of the stable homodimerization of PRELP Q155E. PRELP, proline/arginine-rich end leucine-rich repeat protein.

To address this hypothesis, we prepared recombinant proteins of the Q155R single mutant, which has an opposite charged mutation to Q155E, and the Q155E/R72A double mutant, in which the E155 residue would not associate with the R72 residue (Fig. S12). Analytical SEC showed that these mutations promoted monomerization compared with Q155E (Fig. 6*B*). This result strongly suggests the importance of the E155-R72 interaction for Q155E homodimerization. In particular, the Q155R mutation significantly disrupted PRELP homodimerization, even when compared with WT (Fig. 4*C*). These findings suggest that the partially dissociated Q155E homodimer is likely stable due to the interchain interaction between E155 and R72 residues and therefore resistant to being completely dissociated into monomers (Fig. 6*C*). On the other hand, mass photometry analysis of PRELP Q155E prepared in the buffer containing different salt concentrations did not show significant changes in the monomer-dimer ratio (Fig. S13), suggesting that factors other than the electrostatic interaction between E155 and R72 residues may also contribute to Q155E homodimerization.

### Significance of PRELP homodimerization for its interactions with target proteins

PRELP reportedly functions *via* multiple interactions with diverse proteins (16, 18, 19). Herein, we investigated how the monomer-dimer equilibrium of PRELP affects its interactions with TGFβ1 and IGFI-R using the designed monomeric and dimeric mutants. First, to examine whether the substituted residues in the PRELP mutants are intrinsically critical for the interactions independently of the oligomeric states, we validated the interactions of immobilized PRELP with soluble TGFβ1 and IGFI-R using surface plasmon resonance (SPR) analysis. Once PRELP was immobilized on a sensor chip, measurements could be performed while reducing the influence of shifts in the monomer-dimer equilibrium caused by changes in soluble PRELP concentration. Based on a previous study (18), PRELP mutants were immobilized at approximately 400 and 4000 resonance units (RU) for the interactions with TGFβ1 and IGFI-R, respectively, as it was difficult to observe the IGFI-R binding at low immobilization level probably due to the different accessibility to immobilized PRELP between TGFβ1 and IGFI-R. The results of SPR analysis showed that PRELP Mut5 maintained the interactions with TGFβ1 and IGFI-R (Fig. 7, *A* and *B*), although the binding affinities were a little weaker than those of PRELP WT (Fig. S14*A*, Tables S2 and S3). We did not observe clear binding responses of TGFβ1 with PRELP Q155E immobilized at approximately 400 RU (Fig. 7*C*). Increasing the immobilization level of PRELP Q155E to approximately 4000 RU enabled detection of binding; however, reliable kinetic parameters for the TGFβ1 binding could not be determined due to the fast association and dissociation rates, and the *K*_D_ value calculated from equilibrium curve fitting was much larger than the tested analyte concentration range (Fig. S15, Table S2). In contrast, the binding affinity of PRELP Q155E with IGFI-R was comparable to that of WT (Figs. 7*D* and S14*B*, Table S3). As was seen for Q155E, only weak binding was detected for the interaction of PRELP Q155R with TGFβ1 although the binding affinity would be stronger compared with Q155E (Fig. S16, Table S2), suggesting that the Q155 residue of PRELP is essential for TGFβ1 recognition (*i.e.*, a hot spot residue) independently of the monomer-dimer distribution but does not contribute to IGFI-R binding.

**Figure 7:**
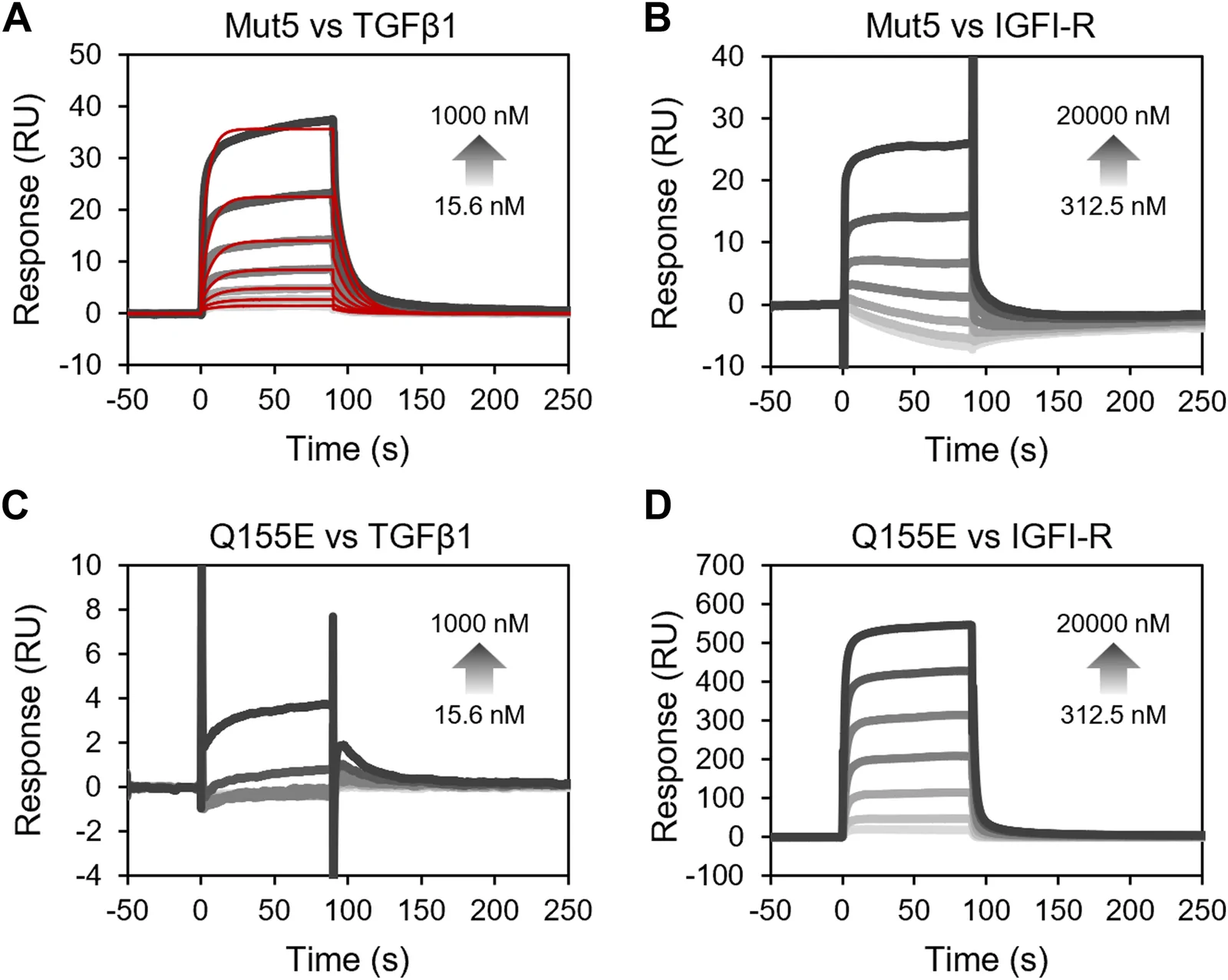
**Influence of mutated residues of PRELP on its interactions with TGFβ1 and IGFI-R.***A* and *B*, direct binding assay of immobilized PRELP Mut5 (Y58D, P60D, F63E, S65E, and A66D) with (*A*) TGFβ1 and (*B*) IGFI-R using SPR. *C* and *D*, direct binding assay of immobilized PRELP Q155E with (*C*) TGFβ1 and (*D*) IGFI-R using SPR. PRELP proteins were immobilized at approximately 400 and 4000 RU for the interactions with TGFβ1 and IGFI-R, respectively. The raw sensorgrams and curve fitting profiles are shown as monochrome and *red lines*, respectively. Line darkness of raw sensorgrams indicates analyte concentrations, with the darkest line indicating the highest concentration. IGFI-R, insulin-like growth factor I receptor; PRELP, proline/arginine-rich end leucine-rich repeat protein; TGFβ1, transforming growth factor β1.

Next, we used nonimmobilized, soluble PRELP mutants and ELISAs to investigate the influence of the monomer-dimer equilibrium of PRELP on its interactions with TGFβ1 and IGFI-R. TGFβ1 or IGFI-R were coated on microtiter wells, and the concentration-dependency of interactions with soluble PRELP WT, Mut5, and Q155E was evaluated. We found that soluble PRELP Mut5 had apparently stronger interactions with TGFβ1 and IGFI-R compared with WT (Fig. 8, *A* and *B*). In contrast, the interaction between soluble PRELP Q155E and IGFI-R was weaker than that of WT (Fig. 8*B*). Although the binding responses of soluble PRELP Q155E to TGFβ1 were also substantially decreased compared with WT (Fig. S17), given that the Q155E mutation would drastically weaken the PRELP-TGFβ1 interaction in the SPR analysis, this result was likely due not solely to the difference in monomer-dimer distributions, but also to the intrinsic loss of a hot spot residue Q155. These results suggest that reversible, concentration-dependent PRELP homodimerization moderates the interactions of PRELP with TGFβ1 and IGFI-R.

**Figure 8:**
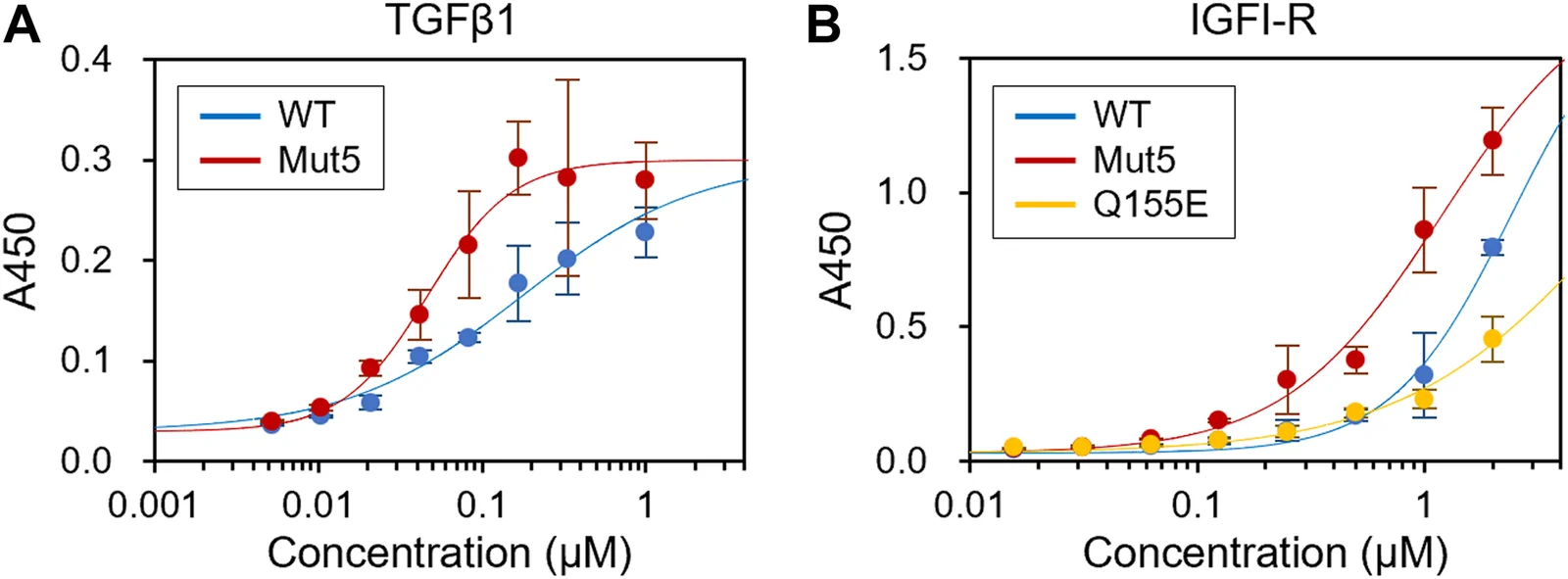
**Contribution of PRELP homodimerization to its interactions with TGFβ1 and IGFI-R.***A* and *B*, ELISA. The wells were coated with (*A*) TGFβ1 or (*B*) IGFI-R, and the bindings of soluble PRELP WT (*blue*), Mut5 (Y58D, P60D, F63E, S65E, and A66D) (*red*), and Q155E (*yellow*) were measured. Bound PRELP was detected with anti-DDDDK-tag mAb HRP-DirecT, and the absorbance at 450 nm was measured after the TMB reaction. IGFI-R, insulin-like growth factor I receptor; PRELP, proline/arginine-rich end leucine-rich repeat protein; TGFβ1, transforming growth factor β1.

### Influence of PRELP homodimerization on tumor cell growth suppression

We previously reported that PRELP suppressed the growth of A549 lung carcinoma cells (19). To test whether PRELP homodimerization affects this tumor suppressive role, we conducted a cell growth assay using a range of concentrations of recombinant PRELP WT, Mut5, or Q155E. The results showed that all of these PRELP constructs significantly suppressed the cell growth of A549 cells at 4 μM protein concentration (Fig. S18), which suggests that PRELP homodimerization did not affect its tumor suppressive function.

## Discussion

The oligomeric propensity of SLRPs and its functional significance have been investigated previously. For example, studies have shown that class I SLRPs decorin and biglycan form homodimers (4, 5, 6), while class II SLRPs osteomodulin and fibromodulin exist as monomers in solution (7, 8). However, our analytical SEC, SEC-MALS, and mass photometry data revealed that PRELP, which is a class II SLRP, is in a concentration-dependent equilibrium of monomers and homodimers (Fig. 1). Furthermore, the SAP calculation and analytical SEC in the presence of detergents suggested that the hydrophobic surface is important for homodimerization of PRELP (Fig. 2). Compared with the results of SAP calculation using crystal structures of bovine decorin and biglycan (Fig. S3), PRELP clearly had a larger hydrophobic patch at the N-terminus in the concave surface of the LRR domain, which is predicted to form the dimer interface of PRELP. Scott *et al.* highlighted the putative importance of a phenylalanine residue sandwiched between two histidine aromatic rings for decorin homodimerization, which is conserved in class I SLRPs but not in class II SLRPs including PRELP, as well as the contribution of an extensive network of hydrogen bonds, salt bridges, and additional hydrophobic interactions between the decorin monomers (4). These observations suggest that PRELP has a distinctive molecular mechanism for dimerization that is driven predominantly by hydrophobicity.

To design monomeric PRELP constructs, we introduced hydrophilic, charged mutations on the hydrophobic dimer interface of PRELP, and we confirmed that the mutations successfully disrupted homodimerization (Figs. 3 and 4). Loss of noncovalent bonds, which are critical for dimerization, due to alanine mutations or disturbing the shape complementarity of the dimer interface *via* substitution with bulky residues would also be effective strategies for disrupting dimerization. However, because exposure of the hydrophobic dimer interface may cause aggregation, as supported by large proportions of void fraction in the SEC profile of monomeric PRELP mutants (Fig. S6), simply weakening the interactions between monomers could significantly compromise colloidal stability, possibly leading to difficulty in production as a soluble protein. For these reasons, we chose to eliminate the surface hydrophobicity based on the SAP calculation, which was an effective approach for inhibiting hydrophobicity-driven dimerization and also for improving colloidal stability of the dissociated monomers to avoid aggregation. It should be noted that the charged mutations may cause electrostatic repulsion, and it is difficult to fully disentangle the direct effects on hydrophobicity from other energetic contributions upon the mutations.

We also serendipitously identified a dimeric PRELP Q155E mutant. Although PRELP Mut5 was predominantly monomeric, Mut6, which only has the additional Q155E mutation, showed a substantially increased dimer population (Fig. S5). This suggests a stronger effect of Q155E mutation on PRELP homodimerization than the other five mutations (Y58D, P60D, F63E, S65E, and A66D). MD simulation suggested that the E155 residue as a monomer tightly associates with the R72 residue in the other monomer after partial dimer dissociation (Fig. 6*A*), and this observation was supported by *in vitro* mutation analysis (Fig. 6*B*). Since no ionic strength-dependent changes in the Q155E dimerization were observed (Fig. S13), it remains unclear whether the E155-R72 electrostatic interaction plays a critical role in the rigid dimerization. However, given that the single Q155R mutation enhanced monomerization, the Q155 residue may act as a switch that regulates the monomer-dimer equilibrium of PRELP. It should be also noted that the partially dissociated homodimer of PRELP Q155E observed in the MD simulation could merely be trapped in an energetic local minimum.

A limitation of this study to investigate the molecular mechanism of PRELP homodimerization is that the computational analyses were performed using homology modelling structures since an experimentally determined PRELP structure is not currently available. Although the models were constructed based on structurally characterized SLRPs, uncertainty in the local structural features may affect mechanistic interpretations at residue level. Nevertheless, the overall distribution of hydrophobic surface identified by the SAP calculation was reproduced using an AlphaFold3-predicted PRELP structure (Fig. S4), supporting the robustness of our central conclusion on the identification of hydrophobic dimer interface. Future determination of the PRELP structure will be necessary to validate the detailed mechanistic insights into PRELP homodimerization proposed in this study.

Our investigations indicated that PRELP is in equilibrium between monomers and dimers and that this reversible homodimerization modulates the target binding properties of PRELP (Fig. 8). In many LRR proteins, the concave surface and adjacent loops are most commonly used for target binding (31), and previous studies also indicated that SLRPs, including decorin and osteomodulin, interact with collagen *via* the concave surface (6, 8, 9, 10). Consistent with these currently accepted concepts, our SPR results highlighted the importance of the Q155 residue of PRELP for the interaction with TGFβ1, although the mutations in this residue did not affect IGFI-R binding (Figs. 7 and S16, Tables S2 and S3). This hot spot residue is located on the LRR concave surface, which forms the dimer interface. Therefore, PRELP likely interacts with TGFβ1 as a monomer, and PRELP dimerization masks the Q155 residue and suppresses the TGFβ1 interaction. We previously reported that PRELP competitively interacts with TGFβ1 and IGFI-R *via* the LRR1–7 region, which suggests that the binding region of PRELP required for the interaction with TGFβ1 overlaps that for IGFI-R (18). Considering these observations, PRELP as a monomer is likely to multi-specifically recognize TGFβ1 and IGFI-R by utilizing different residues on the shared concave surface of the LRR domain. Moreover, these interactions are moderately suppressed by the reversible homodimerization of PRELP (Fig. 9).

**Figure 9:**
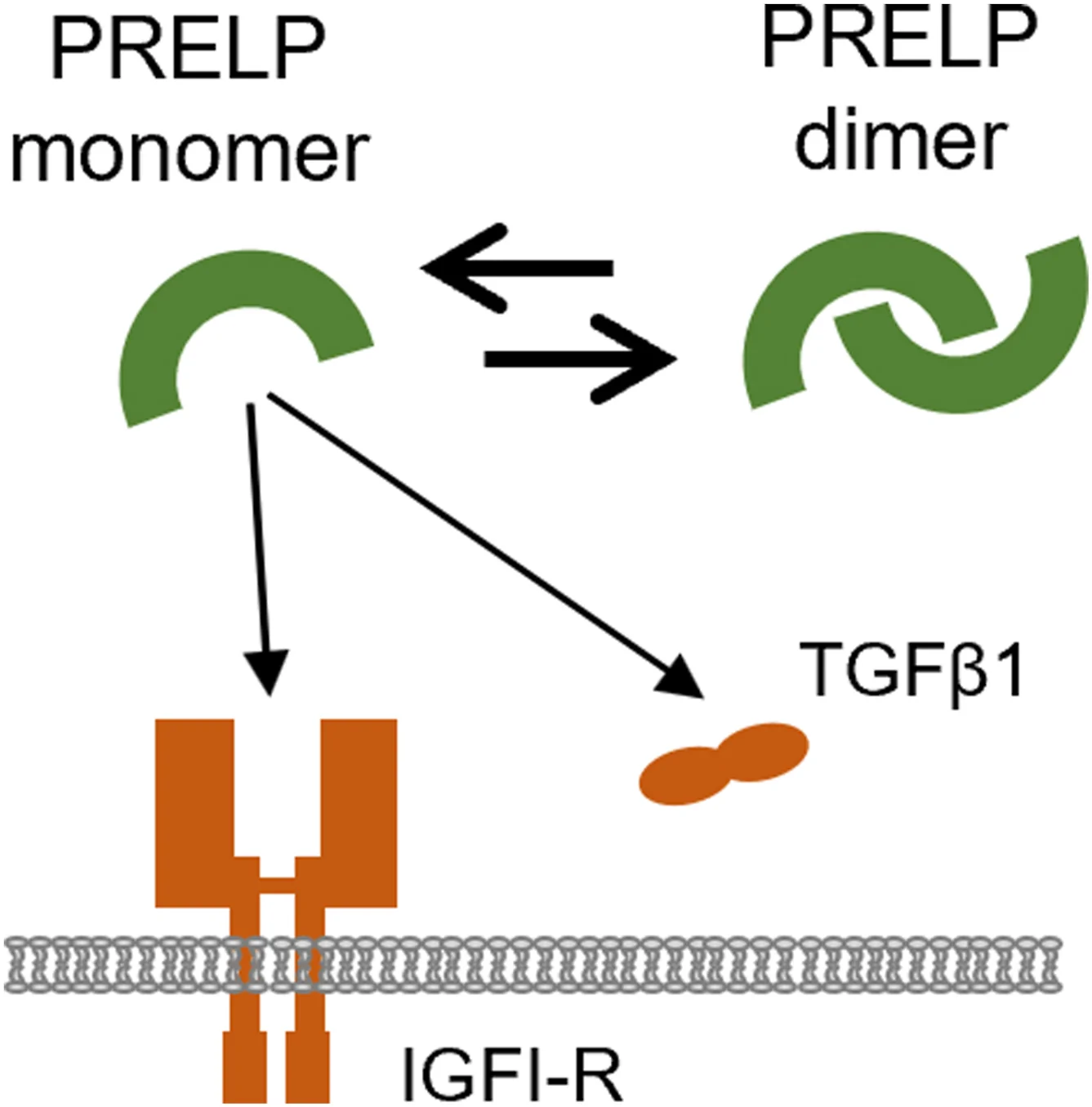
**Model of target interactions *via* the LRR concave surface regulated by PRELP homodimerization.** PRELP forms a reversible homodimer in a concentration-dependent manner *via* the concave surface of LRR domain. Since PRELP also interacts with TGFβ1 and IGFI-R *via* the concave surface as monomer, this reversible homodimerization would modulate the PRELP interactions. IGFI-R, insulin-like growth factor I receptor; LRR, leucine-rich repeat; PRELP, proline/arginine-rich end leucine-rich repeat protein; TGFβ1, transforming growth factor β1. Some graphical elements were adapted from Kosuge *et al*. (18).

For the SPR analysis in this study, it should be noted that relatively high immobilization levels were required in some measurements due to the limited activity and different orientation of immobilized ligands. Therefore, potential avidity effects may not be completely excluded, and the determined interaction parameters should be interpreted with appropriate caution although this would not affect our central conclusions.

Because *PRELP* gene expression is down-regulated in many types of cancer (16, 32), PRELP is expected to function as a key factor in maintaining cellular homeostasis. Our previous studies indicated that PRELP suppresses tumor cell proliferation, changes tumor cell morphology, and alters gene expression in tumor cells, likely through multiple interactions with relatively weak binding affinities (*K*_D_ = 10^–7^–10^–5^ M) (18, 19). Considering that the estimated *K*_D_ value of PRELP homodimerization in the current study was also in the submicromolar range, the concentration-dependent formation of PRELP homodimers would moderately self-repress its target binding ability, and the affinity balance between target binding and homodimerization would ensure that PRELP functions properly even at excessively high protein concentrations. Because PRELP should be unstable in a monomeric state due to the exposure of the hydrophobic surface to solvents, we speculate that it readily forms a relatively stable homodimer when unbound to target proteins to avoid denaturation and aggregation; the dimer then could dissociate into monomers upon target binding. It should be noted that we quantified the monomer-dimer equilibrium of PRELP under simplified solution conditions although PRELP functions in a crowded, highly heterogeneous extracellular matrix environment with extensive interactions with extracellular matrix components, including collagen, perlecan, and fibronectin (18, 33, 34). Such interactions may modulate, compete with, or potentially override PRELP dimerization in the physiological environment. Therefore, the biophysical parameters determined in this study should be regarded as an intrinsic feature of PRELP dimerization under the employed experimental conditions rather than as a direct representation of its behavior *in vivo*. It should be additionally noted that analytical SEC and SEC-MALS measurements were conducted using buffers containing arginine-HCl to avoid nonspecific adsorption of PRELP to the SEC column, which may affect the dimerization propensity of PRELP.

Although we found that PRELP homodimerization regulates its binding with TGFβ1 and IGFI-R, our cell growth assay did not show that dimerization affected the tumor suppressive roles of PRELP (Fig. S18). This observation suggests that these interactions may not significantly contribute to the growth suppression of A549 lung carcinoma cells *in vitro*. The complicated mechanism of action, which includes multiple weak interactions with a wide variety of proteins (19) as well as a concentration-dependent monomer-dimer equilibrium, likely allows PRELP to function in a context- and environment-dependent manner. However, we only tested the suppressive effect of PRELP on a single cell line, and other cell lines which differently express TGFβ1 and IGFI-R should be also tested to further understand the biological significance of PRELP homodimerization. Cell-based experiments which evaluate the alteration of cell signaling and gene expression profile may also provide different results from the cell growth assay.

In conclusion, we found that PRELP is in a concentration-dependent equilibrium between monomers and homodimers and that this equilibrium would be predominantly driven by hydrophobicity. Computational design of monomeric PRELP mutants and *in vitro* interaction analysis showed that the homodimerization of PRELP suppresses its interactions with TGFβ1 and IGFI-R. We propose a model in which the reversible homodimerization of PRELP moderately regulates its molecular functions *via* multi-specific interactions.

## Experimental procedures

### Expression and purification of recombinant PRELP

Recombinant proteins of full-length human PRELP WT and mutants with a FLAG-tag at the C-terminus were prepared following previously described protocols (19). Briefly, the proteins were produced by Mimic Sf9 cells and purified from the culture supernatant using a FLAG-tag affinity column and then by SEC using HiLoad 26/600 Superdex 200 pg or HiLoad 16/600 Superdex 200 pg columns (Cytiva) equilibrated with 20 mM Tris-HCl (pH 8.0), 300 mM NaCl, 400 mM arginine-HCl. The typical final yields from 1 L of culture were approximately 500 to 1000 μg for WT, 250 to 500 μg for Mut5, and 1000 to 2000 μg for Q155E. The protein concentration was determined based on the absorbance at 280 nm measured by a Nanodrop 2000c spectrophotometer (Thermo Fisher Scientific) with the extinction coefficient of 22,265 M^−1^ cm^−1^ for PRELP Y58D, Mut5, and Mut6 and 23,755 M^−1^ cm^−1^ for PRELP WT and the other mutants.

### Analytical SEC

Analytical SEC was performed using 10/300 Superdex 200 Gl columns (Cytiva) equilibrated with 20 mM Tris-HCl (pH 8.0), 150 mM NaCl, 200 mM arginine-HCl at a flow rate of 0.5 ml/min using an AKTA pure and AKTA go system at 4 °C. For each assay, 500 μl of the recombinant PRELP prepared in the running buffer were loaded onto the column, and the absorbance at 280 nm was monitored.

To investigate the influence of additives on the monomer-dimer equilibrium of PRELP, 0.05% (v/v) Tween-20, 0.01% (w/v) DDM, 0.1% (w/v) CHAPS, or 80 μM ANS were added only to the PRELP samples and not to the mobile phase of SEC. The samples were incubated overnight at 4 °C to allow the monomer-dimer equilibrium to be reached and then measured.

To investigate the influence of salt concentration on the monomer-dimer equilibrium of PRELP, the samples were adjusted in 20 mM Tris-HCl (pH 8.0), 500 mM NaCl, 200 mM arginine-HCl, incubated overnight at 4 °C, and then loaded to the column equilibrated with the same buffer containing 500 mM NaCl.

### SEC-MALS

SEC-MALS was performed using 5/150 Superdex 200 Gl columns (Cytiva) equilibrated with 20 mM Tris-HCl (pH 8.0), 150 mM NaCl, 200 mM arginine-HCl at a flow rate of 0.2 ml/min using an inline DAWN8+ MALS detector (Wyatt Technology), a UV detector (Shimadzu), and a refractive index detector (Shodex) at room temperature. For the analysis, 50 μl aliquots of the recombinant PRELP prepared at a range of concentrations were loaded onto the column. Analysis was performed using ASTRA software (Wyatt Technology), and the protein molecular mass was determined. The concentration at the end of the chromatographic column was measured based on UV detection. All detectors were calibrated using bovine serum albumin (BSA) (Sigma-Aldrich).

### Mass photometry

Mass photometry analysis was performed using a TwoMP system (Refeyn) at room temperature.

To evaluate the time-dependent dissociation of PRELP dimer upon dilution, the droplet dilution method was used. The focus was adjusted in 10 mM Hepes (pH 7.5), 150 mM NaCl, and 500 nM of PRELP were 10-fold diluted into the buffer (to 50 nM total monomer concentration). Immediately after loading the samples, data were consecutively recorded five times for 1 min each time. The counts of molecules that landed on the glass surface and the interference signal of light scattering by the molecules were measured. The protein mass was calculated from the signal using a calibration curve with BSA monomers and dimers. The recorded data were split and analyzed in 30 s segments. To evaluate the difference in the monomer-dimer ratio among PRELP mutants, the buffer-free method was used. Prior to the measurements, the samples were adjusted to 50 nM in 10 mM Hepes (pH 7.5), 150 mM NaCl and incubated at room temperature for 1 h to allow the monomer-dimer equilibrium to be reached. Immediately after loading the incubated samples without dilution, data were recorded for 1 min. For measurements to verify the influence of salt concentration on the monomer-dimer ratio of PRELP Q155E, the sample was adjusted to 50 nM in 10 mM Hepes (pH 7.5), 500 mM NaCl and measured after 1h incubation. Using data for the molecule counts of monomers and dimers, the *K*_D_ of dimerization was calculated based on a previously reported formula (35) as follows:

$$K_{D}=\frac{{\left[Monomer\right]}^{2}}{\left[Dimer\right]}=\frac{N_{M}^{2}}{N_{D\;}\left(2N_{D}+N_{M}\right)}\times 50\;\text{nM}$$

where N_M_ and N_D_ are the measured counts of monomer and dimer molecules, respectively. The *K*_D_ of dimerization was calculated in three to five technical replicates using the above formula, and the average value with standard deviation was calculated.

### SAP calculation

SAP was calculated following the methodology described in a previous study (26) but using an in-house script based on CHARMM software (36) as described previously (37) without MD simulations. For the calculation, we used a model structure of human PRELP monomer (amino acid residues 55–361) from the SWISS-MODEL repository (27), which is modeled based on osteomodulin (PDB: 5YQ5) by sequential homology, and a monomeric chain from crystal structures of bovine decorin (PDB: 1XKU) and biglycan (PDB: 2FT3). The SAP value for each amino acid residue was calculated by summing the SAP values for all component atoms. The SAP mapped structure was visualized using UCSF Chimera (38).

### Mutational design using rosetta

To design monomeric PRELP mutants, we selected 15 amino acid residues that had SAP values > 2 in WT. We then used Rosetta software (29) (version 2018.33.60351) to calculate the energy differences between WT and a single mutant in which the selected residues were replaced with a charged amino acid. Using a model structure of human PRELP monomer (amino acid residues 55–361) in the SWISS-MODEL repository, ΔΔG values upon mutation were calculated using the Cartesian_ddg protocol (39, 40) with the REF2015 scoring function (40). Five independent simulations were conducted for each mutation, and the average ΔΔG values were calculated.

### CD

CD spectra were measured using J-820 or J-1500 spectropolarimeters (Jasco) using the sample prepared at 2 μM in 10 mM Hepes pH 7.5, 150 mM NaCl at 20 °C. CD measurements were performed at a scanning speed of 20 nm/min by continuous scanning from 260 to 200 nm using a 1 mm path-length quartz cell. Five scans were accumulated, and the collected data were analyzed using Spectra Analysis software (Jasco).

### MD simulation

MD simulations of PRELP dimers were performed using GROMACS 2018.6 (41) with the CHARMM36m force field (42). The dimer structures of human PRELP WT, Mut5, and Q155E (amino acid residues 55–361) modeled using MODELLER 10.2 (30) based on the crystal structure of bovine biglycan dimer (PDB: 2FT3) were used as initial structures. Protonation states of amino acid residues at pH 7 were assessed using PDB2PQR (43). Solvation of the structures was performed with TIP3P water (44) in a rectangular box so that the minimum distance to the edge of the box was 15 Å under periodic boundary conditions. The protein charge was neutralized with added Na or Cl, and additional ions were added to imitate a salt solution of concentration 0.15 M. Each system was energy-minimized for 5000 steps, equilibrated in the canonical (NVT) ensemble at 298 K for 200 ps and subsequently in the isothermal–isobaric (NPT) ensemble at 298 K for 200 ps. A further production run was performed with the NPT ensemble at 298 K for 400 ns. The time step was set to 2 fs throughout the simulations. Each simulation was conducted three times (run-1, -2, -3). All trajectories were analyzed and visualized using GROMACS tools and UCSF Chimera. RMSD of the Cα atoms was computed after superposition of the Cα atoms of each dimer structure during the simulations, with reference to the initial structure of the production runs. Distances between specific atoms during the simulations were also measured and analyzed.

### Expression and purification of recombinant TGFβ1 and IGFI-R

Recombinant human TGFβ1 was prepared as described previously (18). Briefly, latent TGFβ1 proteins were produced by ExpiCHO cells and purified by Ni-NTA affinity chromatography. After the activation with 100 mM acetic acid, mature TGFβ1 proteins were purified by cation exchange chromatography. The ectodomain of recombinant, soluble human IGFI-R was prepared as described previously (19). Briefly, the proteins were produced by ExpiCHO cells and purified by Ni-NTA affinity chromatography and finally by SEC. The protein concentration was determined based on the absorbance at 280 nm measured by a Nanodrop 2000c spectrophotometer with the extinction coefficient of 57,840 M^−1^ cm^−1^ for TGFβ1 and 298,810 M^−1^ cm^−1^ for IGFI-R.

### SPR

SPR measurements were performed using a Biacore T200 instrument (Cytiva) following previously described methods (18). Recombinant PRELP proteins were immobilized using an amine coupling method on a CM5 Biacore sensor chip at around 400 and 4000 RU for the interactions with TGFβ1 and IGFI-R, respectively. PRELP Q155E and Q155R were immobilized also at around 4000 RU for the interactions with TGFβ1. Recombinant TGFβ1 (15.6–1000 nM) or IGFI-R (312.5–20,000 nM) was injected into the sensor chip at a flow rate of 30 μl/min. The assay was carried out in 10 mM Hepes (pH 7.5), 150 mM NaCl, containing 0.05% (v/v) Tween-20 at 15 °C. For the TGFβ1 assay, 50 mM arginine-HCl was also added to the buffer. A regeneration procedure was performed at the end of each cycle with 1 M arginine-HCl (pH 4.4). The data were analyzed using BIAevaluation software (Cytiva). Kinetic parameters and dissociation constants were obtained by global fitting or equilibrium curve fitting.

### ELISA

ELISA measurements were performed following previously described methods (18). Microtiter wells were coated with recombinant TGFβ1 or IGFI-R at 10 μg/ml in 10 mM Hepes pH 7.5, 150 mM NaCl overnight at 4 °C. The wells were washed three times with washing buffer (10 mM Hepes pH 7.5, 150 mM NaCl, 0.05% (v/v) Tween-20) and blocked with washing buffer containing 10% (w/v) BSA at room temperature for 2 h. The wells were washed three times and then incubated with a range of concentrations of recombinant PRELP proteins in the washing buffer containing 1% (w/v) BSA at room temperature for 1 h. After washing three times, bound PRELP was detected with anti-DDDDK-tag mAb HRP-DirecT (MBL) in the washing buffer containing 5% (w/v) BSA at room temperature for 1 h. The wells were washed three times and developed with TMB soluble reagent (ScyTek Laboratories). The reaction was stopped by adding TMB stop buffer to the wells, and the plates were read at 450 nm using an ARVO-X3 instrument (PerkinElmer).

### Cell growth assay

The cell growth assay was conducted as described previously (19). Briefly, A549 cells (RIKEN) were cultured in 48-well plates in DMEM containing 10% (v/v) fetal bovine serum and different concentrations of recombinant PRELP WT, Mut5, or Q155E for 96 h at 37 °C and 5% CO_2_. Six (for 0 nM) or three (for the other concentrations) wells were prepared for each concentration. Cell viability was assessed by the addition of Cell Count Reagent SF (Nacalai Tesque) and measurement of absorbance at 450 nm using an ARVO-X3 instrument. For the 0 nM conditions, measured values exceeding 1.5 times the interquartile range were presumed to result from operational errors and therefore excluded from the analysis as outliers.

## Data availability

The data obtained in this study are available from the corresponding author (tsumoto@bioeng.t.u-tokyo.ac.jp) upon reasonable request.

## Supporting information

This article contains supporting information (18).

## Conflict of interest

The authors declare that they have no conflicts of interest with the contents of this article.
