# Supplementary material for: Reversible homodimerization of a small leucine-rich proteoglycan PRELP modulates the interactions with TGFβ1 and IGFI-R
Source: J Biol Chem. 2026 Sep 8;302(10):113523. doi: 10.1016/j.jbc.2026.113523 (PMC13671038; doi:10.1016/j.jbc.2026.113523)
Supplement: Supplementary file 1 [file mmc1.pdf]

Supporting information for:

Reversible homodimerization of a small leucine-rich proteoglycan PRELP modulates the interactions with TGFβ1 and IGFI-R

Hirofumi Kosuge[1], Makoto Nakakido[1, 2], Daisuke Kuroda[3], Kouhei Tsumoto[1, 2, 4, *]

[1] Department of Bioengineering, School of Engineering, The University of Tokyo, 7-3-1, Hongo, Bunkyo-ku, Tokyo 113-8656, Japan

[2] Department of Chemistry and Biotechnology, School of Engineering, The University of Tokyo, 7-3-1, Hongo, Bunkyo-ku, Tokyo 113-8656, Japan

[3] Department of Biosciences, College of Humanities and Sciences, Nihon University, 3-25-40, Sakurajosui, Setagaya-ku, Tokyo, 156-8550, Japan

[4] The Institute of Medical Science, The University of Tokyo, 4-6-1, Shirokanedai, Minato-ku, Tokyo 108-8639, Japan

[*] Correspondence and requests for materials should be addressed to K.T. (tsumoto@bioeng.t.u-tokyo.ac.jp)

Table S1. Amino acid residues of PRELP with positive SAP values within 55–300 amino acid residues

Residue number [a]	Amino acid residue	SAP value [b]	Notes
63	Phe	12.14	
60	Pro	11.10	
66	Ala	10.76	
68	Tyr	9.08	
58	Tyr	8.61	
110	Trp	8.57	
67	Leu	8.11	
59	Cys	8.09	Forming intramolecular disulfide bond
86	Tyr	7.53	
134	Phe	6.89	
61	Pro	6.42	
62	Asp	6.29	Charged residue
80	Ile	5.10	Convex surface
87	Leu	4.38	
155	Gln	3.53	
84	Ile	3.46	
88	Tyr	2.92	
168	Pro	2.63	Convex surface
65	Ser	2.16	
179	Leu	1.95	
296	Leu	1.74	
64	Pro	1.72	
81	Pro	1.44	Convex surface
203	Leu	1.31	
111	Ile	1.19	
226	Gln	0.87	
135	Leu	0.84	
169	Gly	0.59	Convex surface
216	Met	0.28	Convex surface
205	Gln	0.27	
225	His	0.24	
170	Val	0.05	Convex surface

[a] The residue numbering is based on the mature human PRELP protein sequence, with the N-terminal residue after signal peptide cleavage designated as residue 1.

[b] The SAP value for each amino acid residue was calculated by summing the SAP values for all component atoms.

Table S2. Kinetic parameters of the interaction of TGFβ1 with PRELP mutants [a]

	k_{on} ($\times 10^4$ M^{-1} s^{-1})	k_{off} ($\times 10^{-2}$ s^{-1})	K_D (μM)
PRELP WT [b]	51.3 ± 2.8	19.6 ± 2.3	0.380 ± 0.037
PRELP Mut5	6.00 ± 2.19	6.31 ± 1.16	1.38 ± 0.40
PRELP Q155E	N.D.	N.D.	(125000 ± 63000) [c]
PRELP Q155R	N.D.	N.D.	(8.64 ± 0.60) [c]

[a] At least three independent measurements were carried out, and the average values with standard errors are shown. N.D. indicates "not determined."
[b] Data from our previous study (Kosuge, H. et al. PRELP functions via multiple interactions with intrinsically weak affinity relying on ECM anchoring and remodeling. *Sci Rep* 15, 24634 (2025). https://doi.org/10.1038/s41598-025-09018-7)
[c] The affinities were calculated by equilibrium curve fitting for the SPR results in which PRELP proteins were immobilized at approximately 4000 RU. The calculated K_D values are well outside the tested analyte concentration range (15.6–1000 nM) and therefore of limited quantitative reliability.

Table S3. Affinities of the interaction of PRELP mutants with IGFI-R [a]

	WT [b]	Mut5	Q155E
K_D (μM)	7.09 ± 1.13	22.0 ± 7.9	5.51 ± 0.34

[a] At least three independent measurements were carried out, and the average values with standard errors are shown.
[b] Data from our previous study (Kosuge, H. et al. PRELP functions via multiple interactions with intrinsically weak affinity relying on ECM anchoring and remodeling. *Sci Rep* 15, 24634 (2025). https://doi.org/10.1038/s41598-025-09018-7)

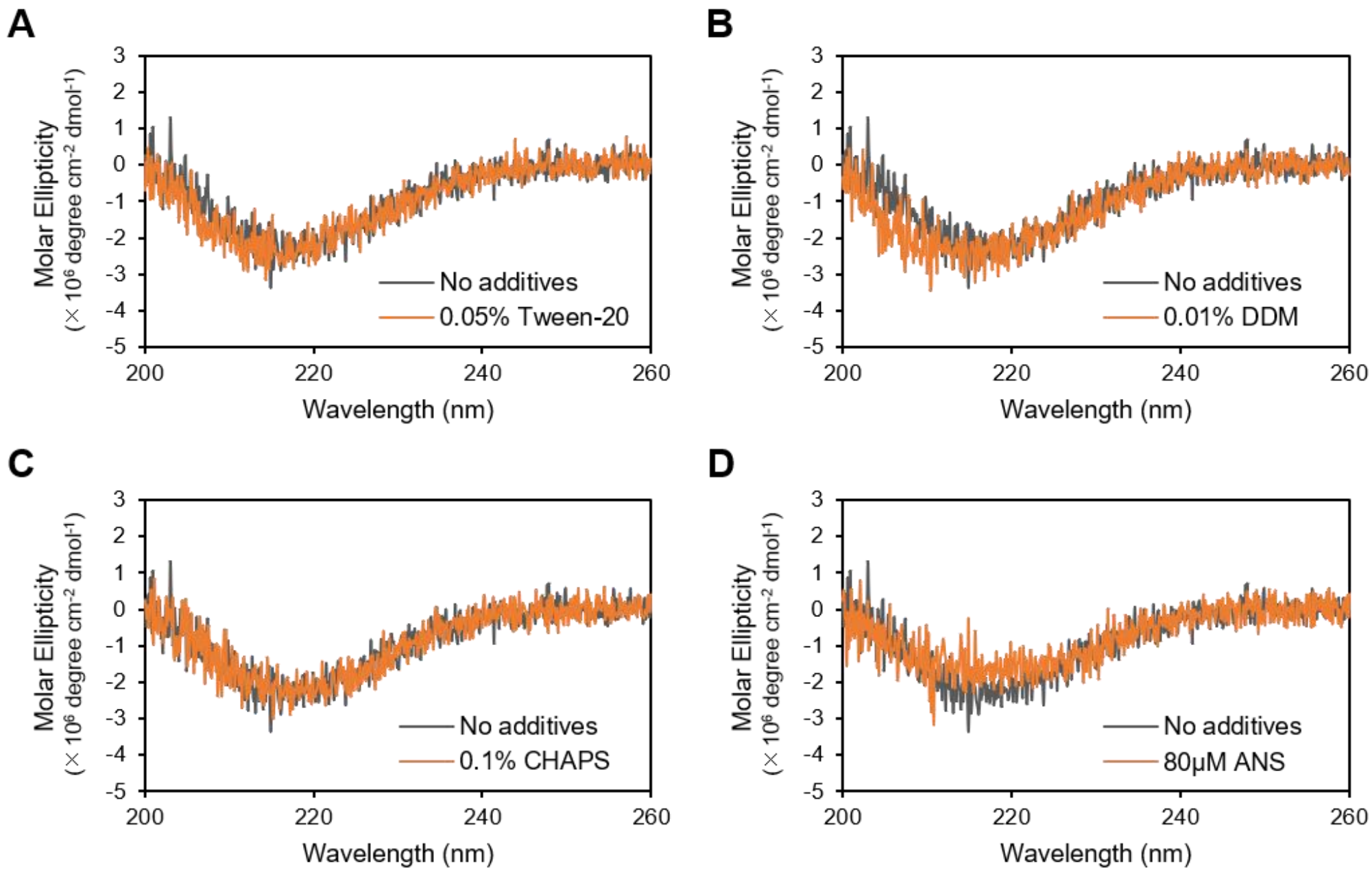

Figure S1. CD measurements of PRELP in the presence of additives. CD spectra of PRELP samples containing (A) 0.05% (v/v) Tween-20, (B) 0.01% (w/v) DDM, (C) 0.1% (w/v) CHAPS, (D) 80 µM ANS. The spectra in the presence and absence of the additives are shown in orange and gray, respectively.

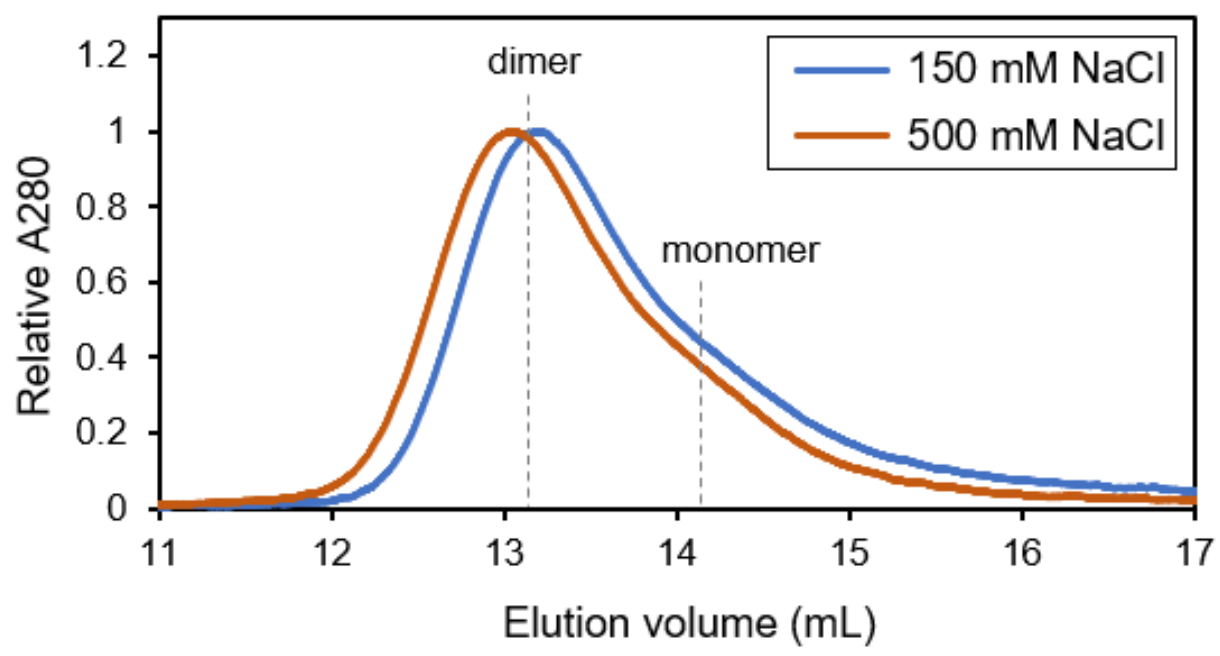

Figure S2. Analytical SEC of PRELP at different salt concentrations. 4 µM of PRELP proteins were injected. The samples and running buffer were adjusted in 20 mM Tris-HCl (pH 8.0), 150 mM NaCl, 200 mM arginine-HCl (blue) or 20 mM Tris-HCl (pH 8.0), 500 mM NaCl, 200 mM arginine-HCl (orange).

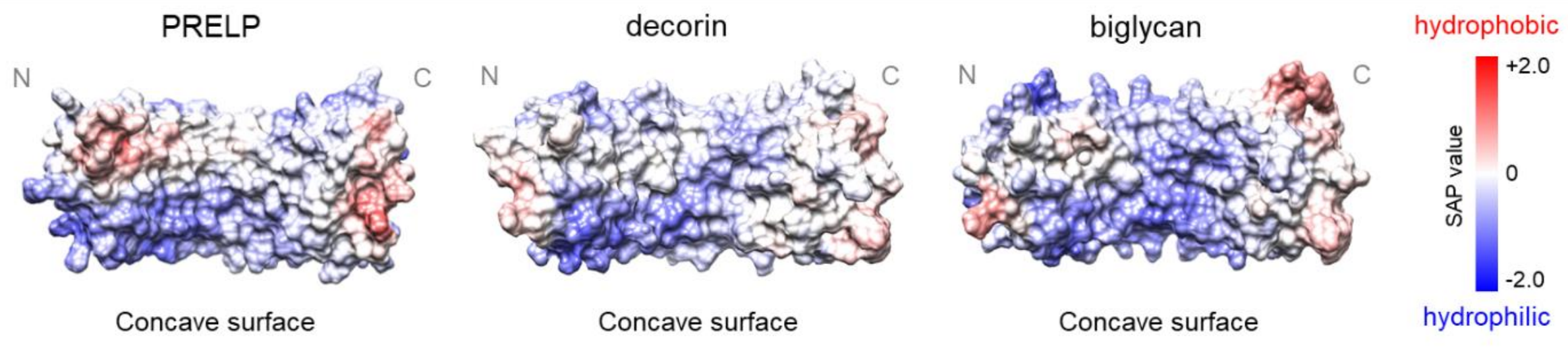

Figure S3. Comparison of the SAP calculation of the human PRELP, bovine decorin, and bovine biglycan monomer. The calculated SAP value of each atom was mapped onto a model structure of PRELP monomer in the SWISS-MODEL repository and crystal structures of decorin (PDB: 1XKU) and biglycan (PDB: 2FT3), and the concave surface of the monomer is shown.

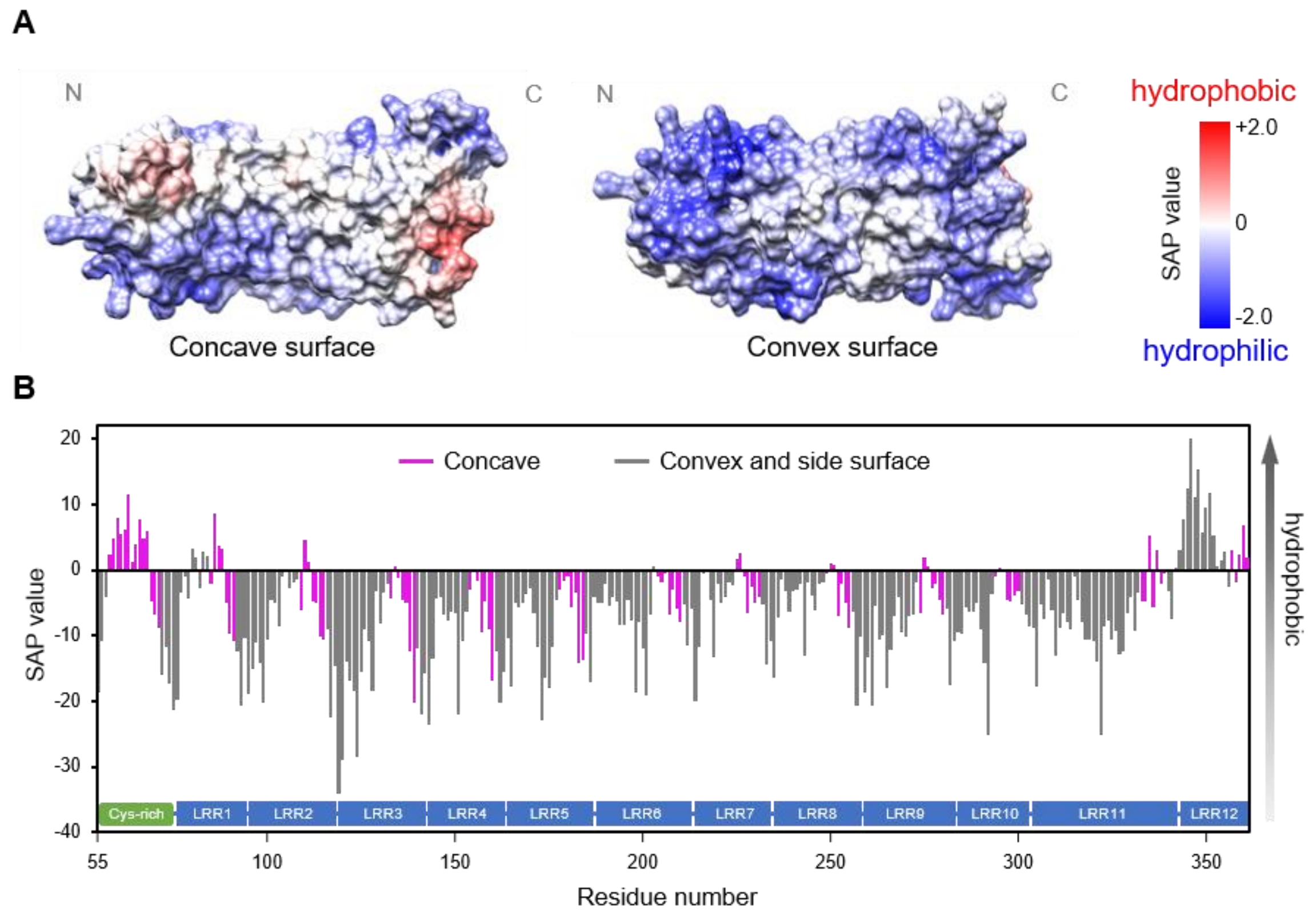

Figure S4. SAP calculation of PRELP using an AlphaFold3-predicted structure. (A) SAP calculation of the PRELP monomer. The calculated SAP value of each atom was mapped onto a model structure of the PRELP monomer predicted by AlphaFold3. (B) SAP value of each amino acid residue of the PRELP monomer; the residues on the concave surface are colored in magenta. The side surface refers to the edge of the horseshoe-shaped LRR domain located between the convex and concave face. The residue numbering is based on the mature human PRELP protein sequence, with the N-terminal residue after signal peptide cleavage designated as residue 1.

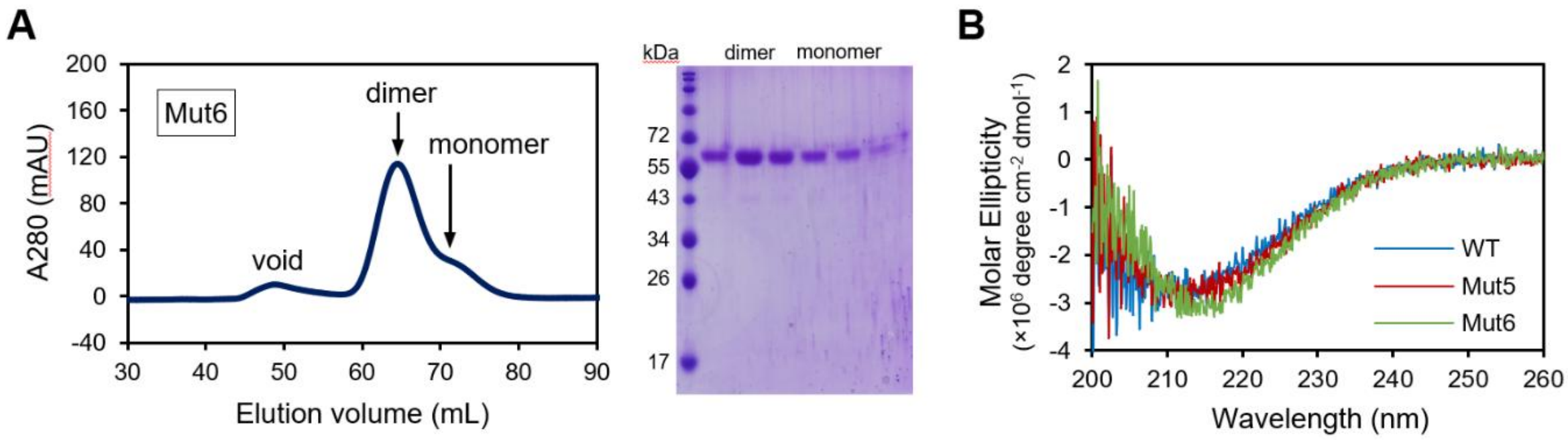

Figure S5. (A) SEC purification profile and SDS-PAGE of PRELP Mut6 (Y58D, P60D, F63E, S65E, A66D, and Q155E); (B) CD spectra of the purified PRELP WT (blue), Mut5 (Y58D, P60D, F63E, S65E, and A66D) (red), and Mut6 (green)

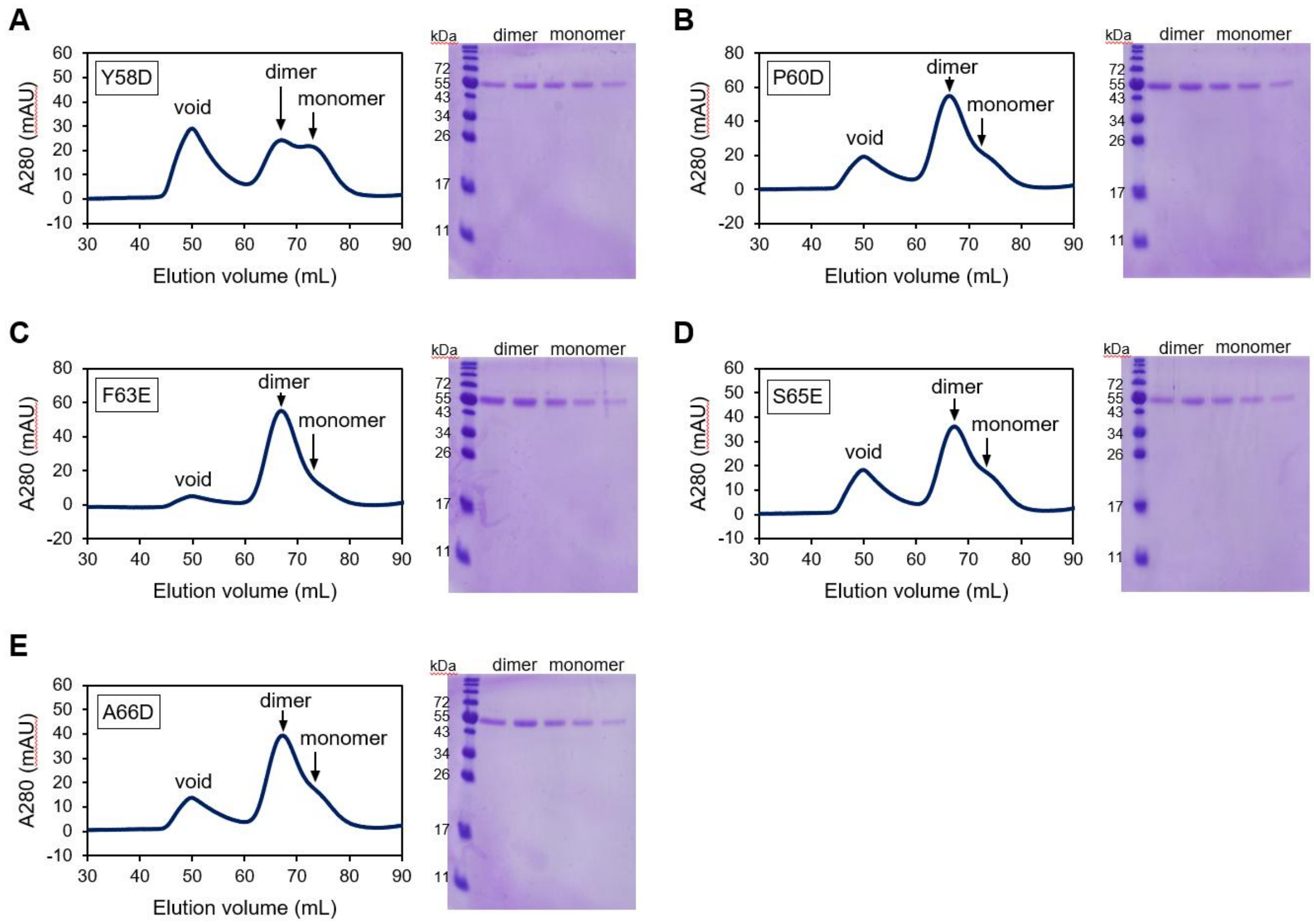

Figure S6. SEC purification profile and SDS-PAGE of PRELP single mutants: (A) Y58D, (B) P60D, (C) F63E, (D) S65E, and (E) A66D

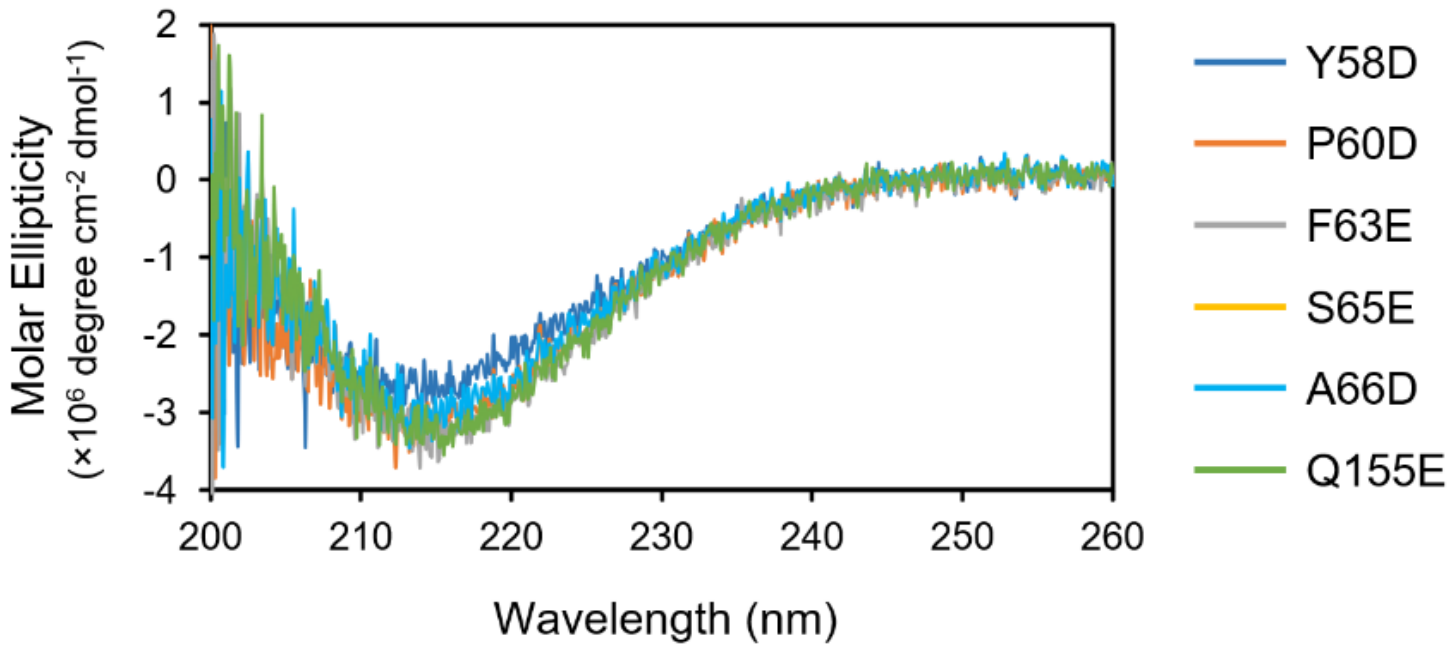

Figure S7. CD spectra of the purified PRELP single mutants

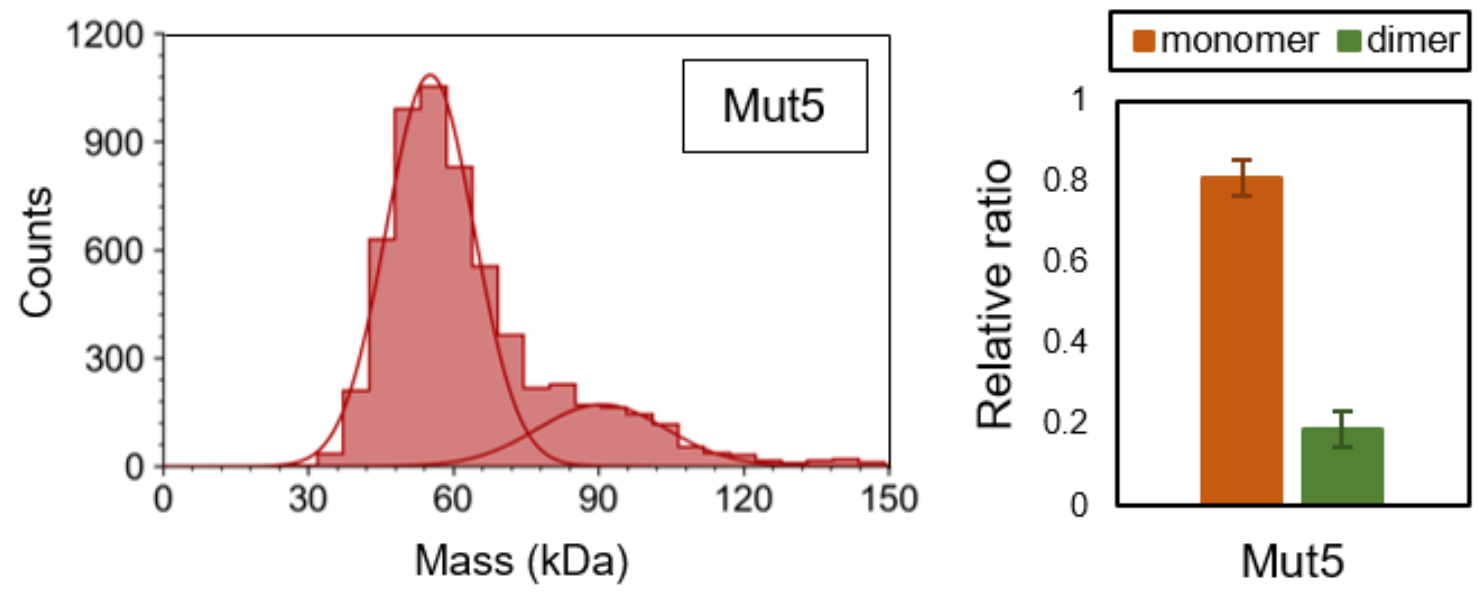

Figure S8. Mass photometry analysis of PRELP Mut5 (Y58D, P60D, F63E, S65E, and A66D). Mass distribution was measured without dilution after the samples were adjusted to 50 nM and incubated for 1 h to allow the monomer-dimer equilibrium to be reached. The average values ± standard deviations of the relative abundance ratio of monomers and dimers in three technical replicates are shown.

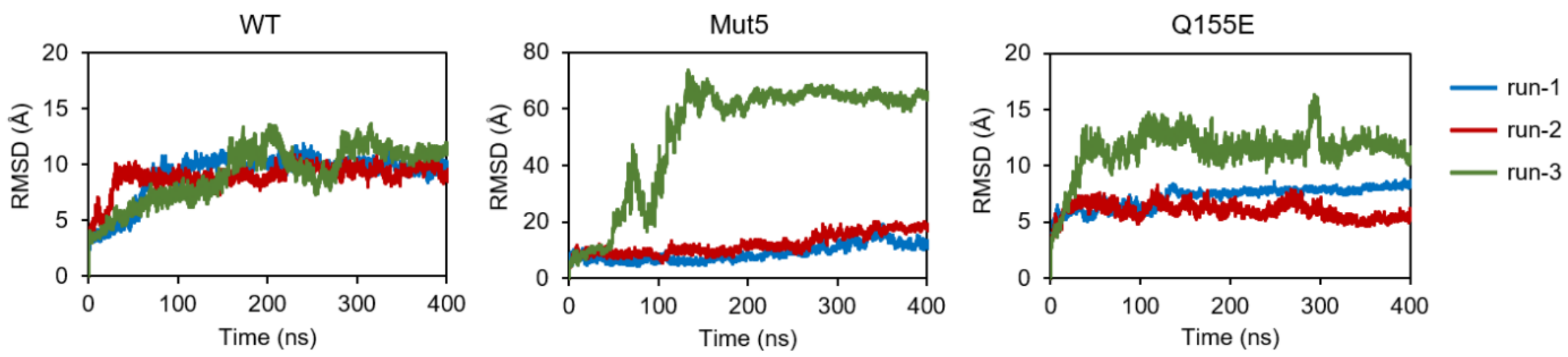

Figure S9. RMSD values of Cα atoms of PRELP dimer WT, Mut5 (Y58D, P60D, F63E, S65E, and A66D), and Q155E between simulation and the initial structure

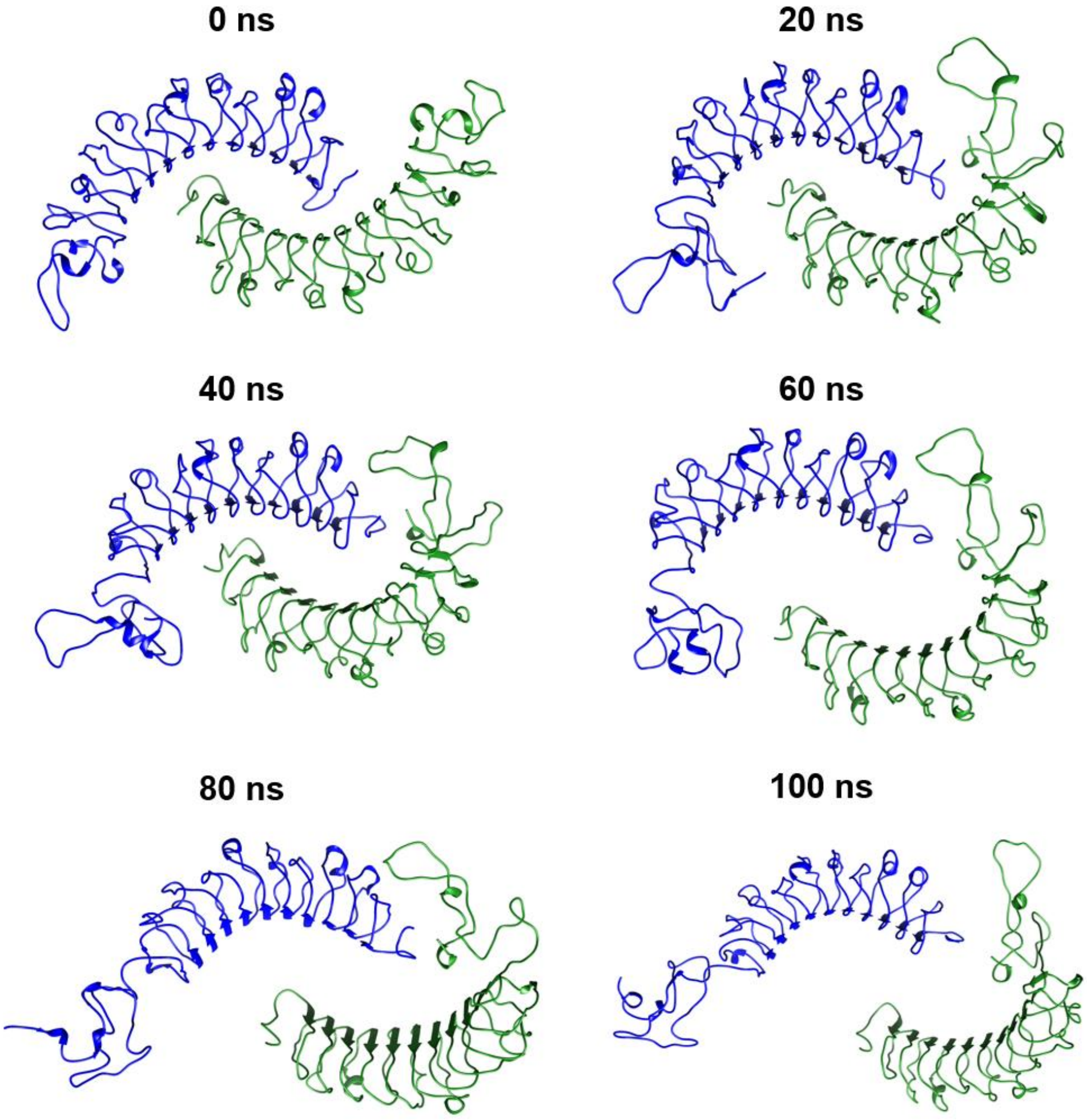

Figure S10. Snapshots of the MD movie in simulation run-3 of the PRELP Mut5 (Y58D, P60D, F63E, S65E, and A66D) homodimer at certain time points; blue and green ribbon models represent monomer chain A and B, respectively.

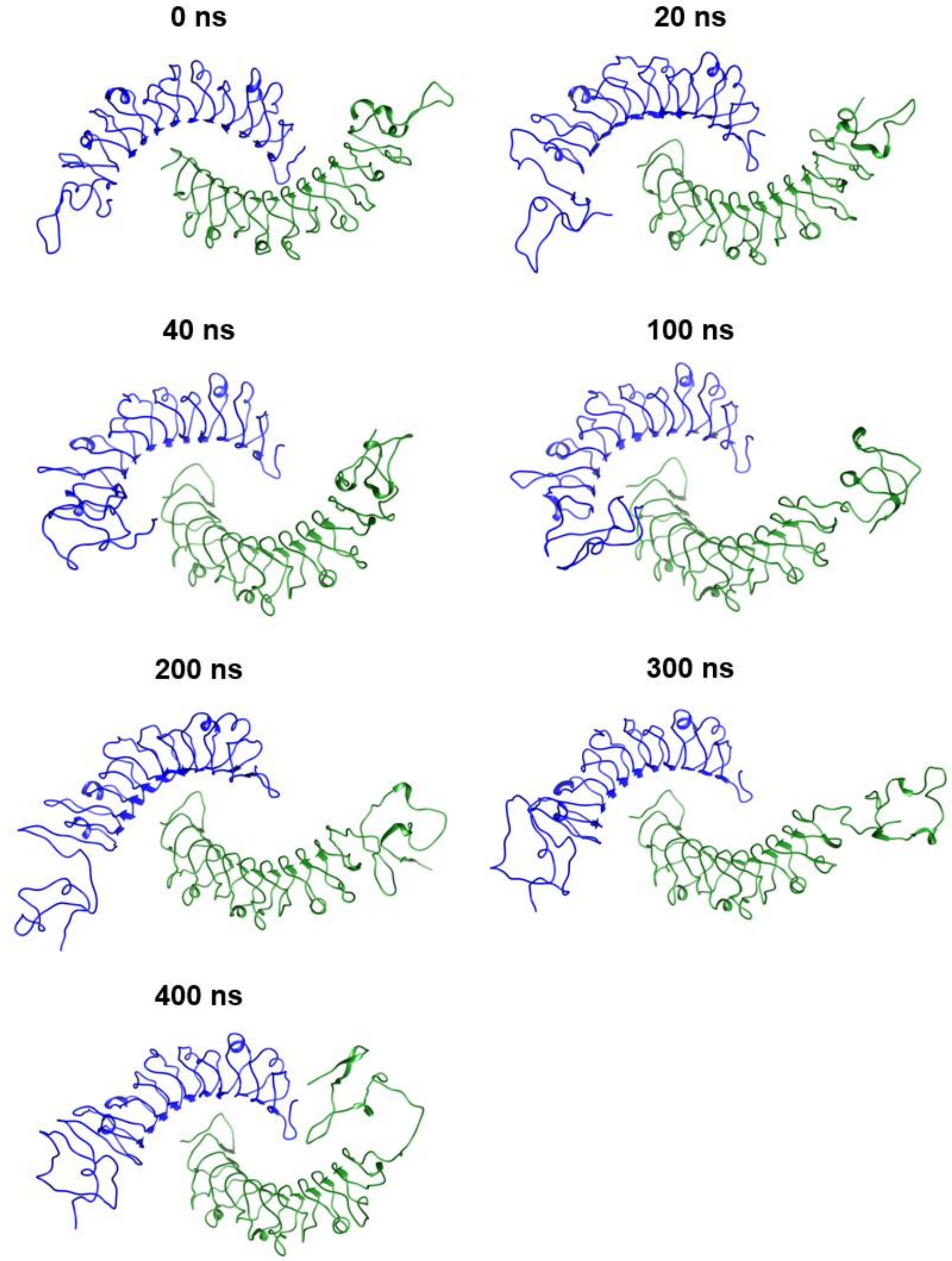

Figure S11. Snapshots of the MD movie in simulation run-3 of the PRELP Q155E homodimer at certain time points; blue and green ribbon models represent monomer chain A and B, respectively.

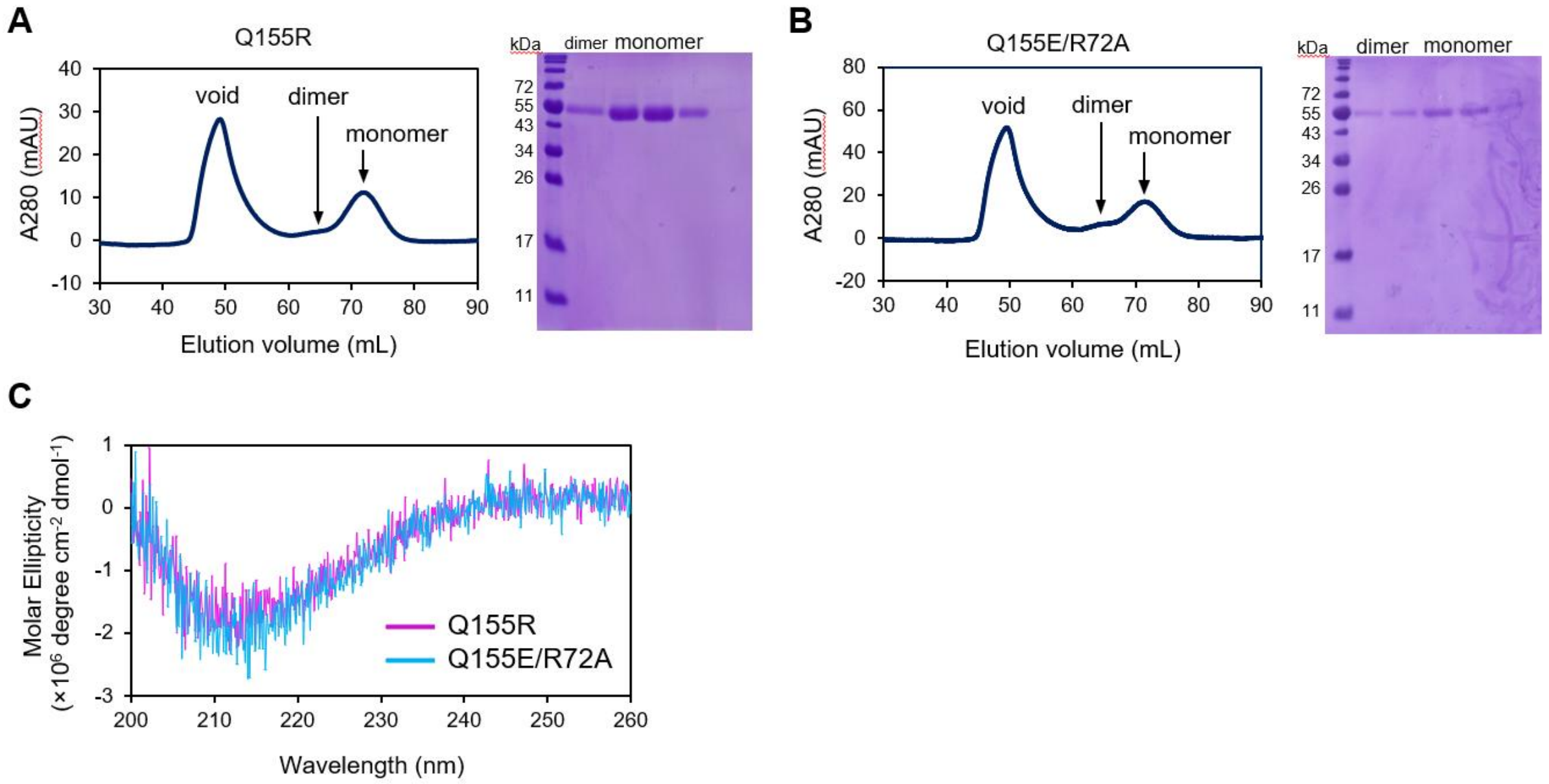

Figure S12. Preparation of recombinant PRELP Q155R single mutant and Q155E/R72A double mutant. (A–B) SEC purification profile and SDS-PAGE of (A) Q155R and (B) Q155E/R72A. (C) CD spectra of the purified PRELP Q155R (magenta) and Q155E/R72A (light blue).

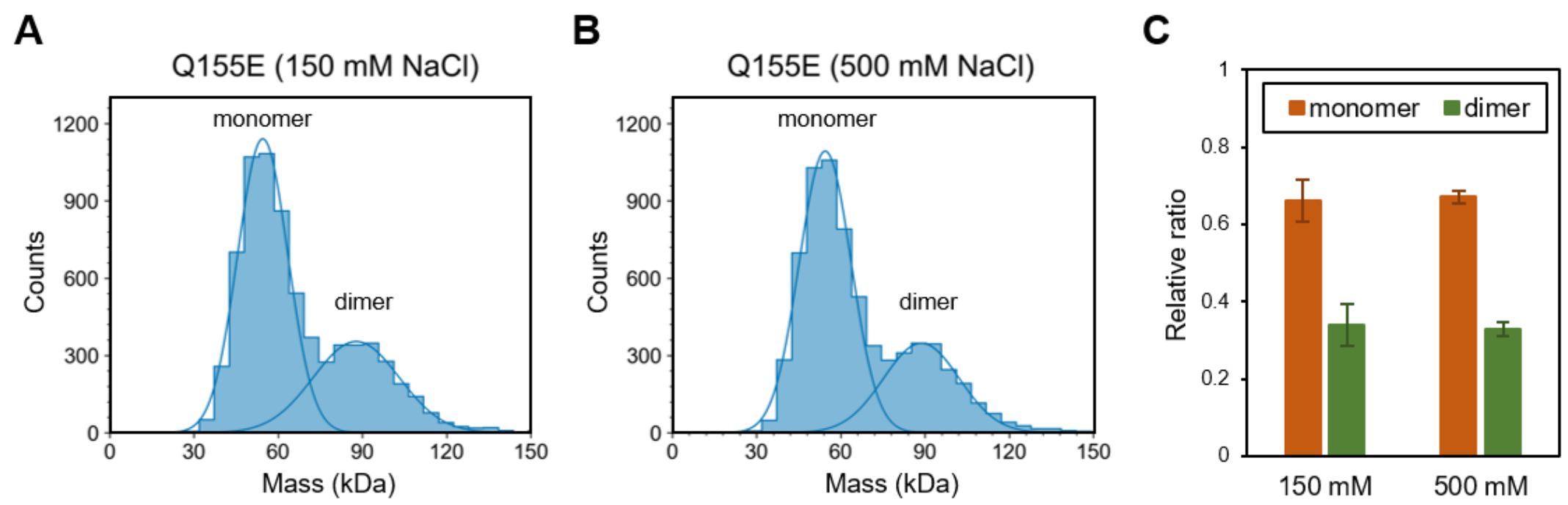

Figure S13. Mass photometry analysis of PRELP Q155E in the buffer containing different salt concentrations. (A-B) Mass distribution. The measurements were performed without dilution after the samples were adjusted to 50 nM in (A) 10 mM HEPES (pH 7.5), 150 mM NaCl or (B) 10 mM HEPES (pH 7.5), 500 mM NaCl and incubated for 1 h to allow the monomer-dimer equilibrium to be reached. (C) Relative abundance ratio of monomers and dimers. The average values $\pm$ standard deviations of five technical replicates are shown.

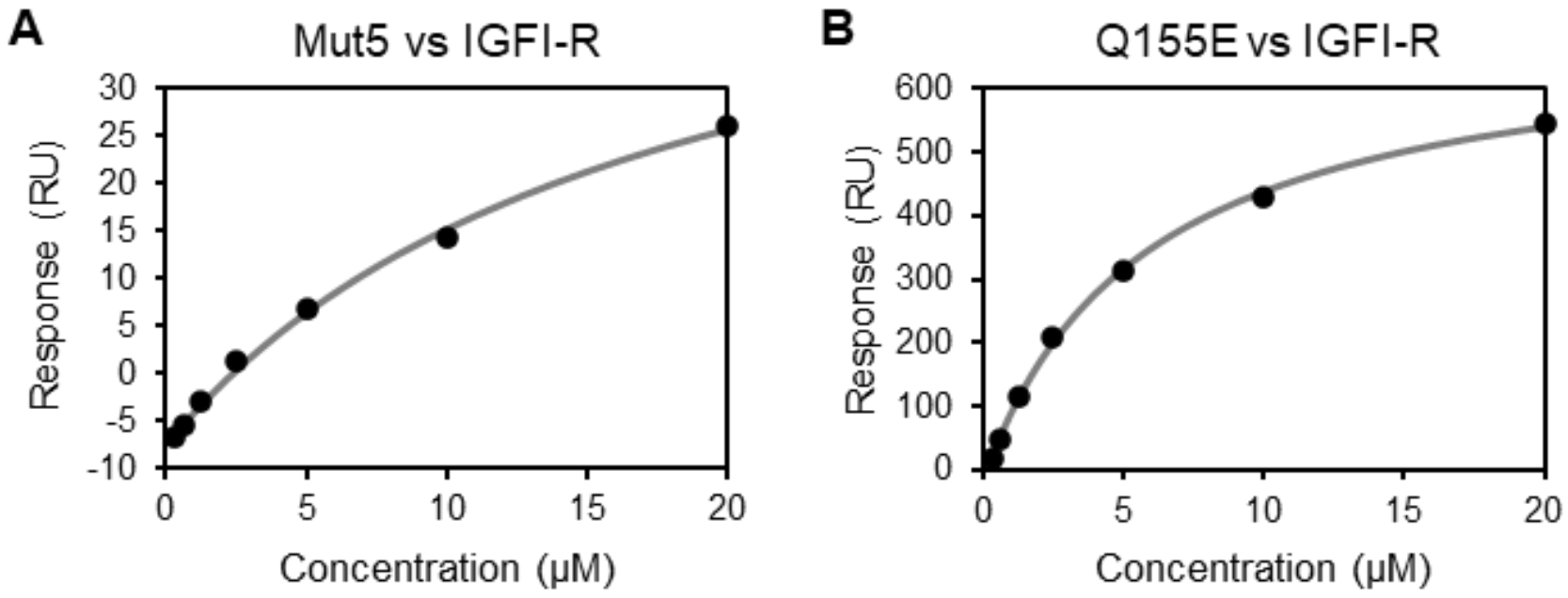

Figure S14. Response units vs. concentration of IGFI-R for the interaction with (A) PRELP Mut5 (Y58D, P60D, F63E, S65E, and A66D) and (B) PRELP Q155E in a direct binding assay using SPR

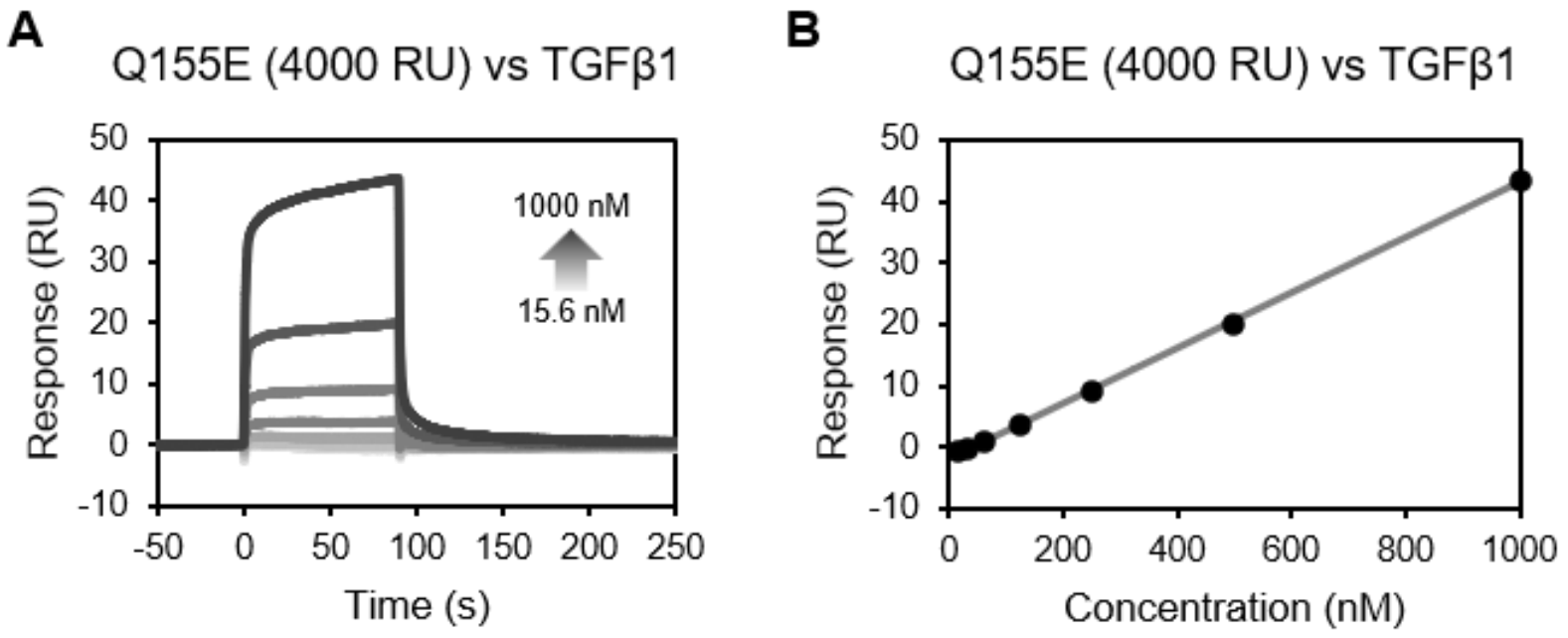

Figure S15. Direct binding assay of TGFβ1 with PRELP Q155E immobilized at approximately 4000 RU using SPR. (A) SPR response vs. time curves. Line darkness indicates analyte concentrations, with the darkest line indicating the highest concentration. (B) Response units vs. concentration of TGFβ1.

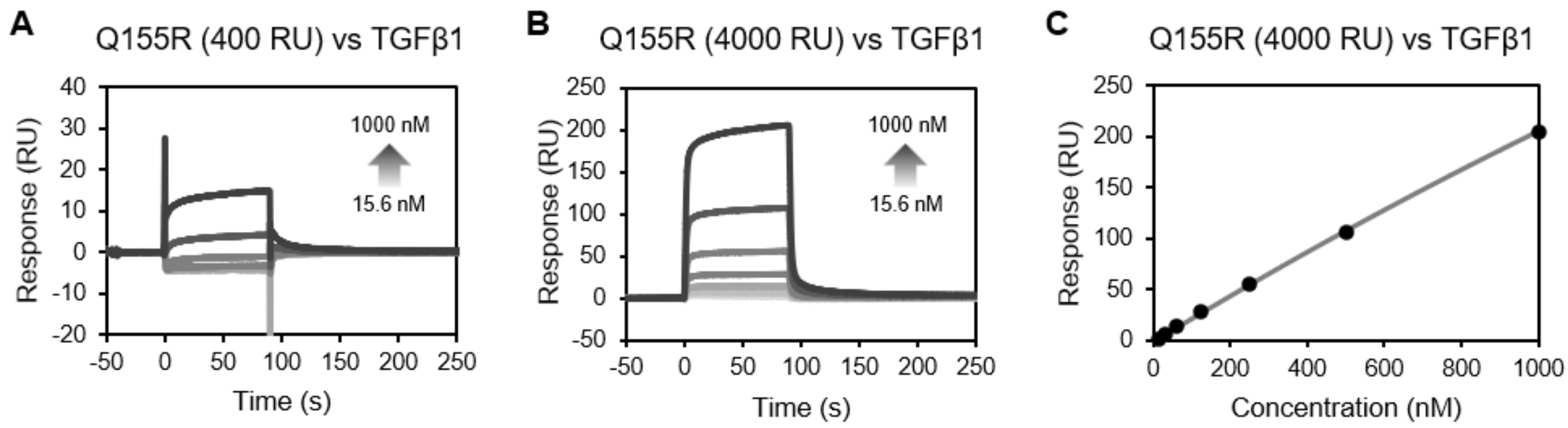

Figure S16. Interaction analysis between PRELP Q155R and TGFβ1. (A-B) Direct binding assay using SPR. PRELP Q155R was immobilized at approximately (A) 400 or (B) 4000 RU. Line darkness indicates analyte concentrations, with the darkest line indicating the highest concentration. (C) Response units vs. concentration of TGFβ1 for the interaction with PRELP Q155R immobilized at approximately 4000 RU.

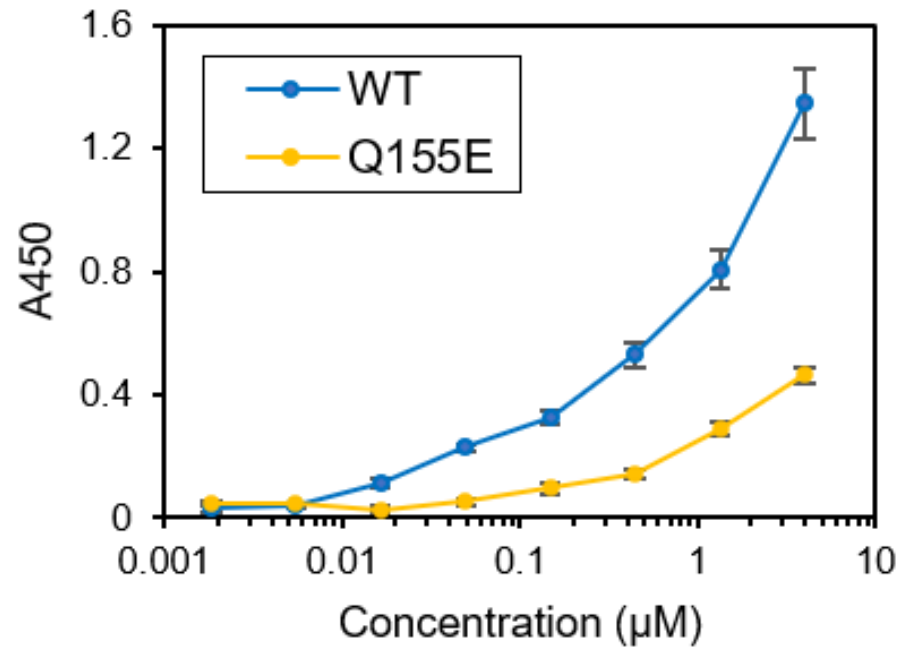

Figure S17. Binding assay between immobilized TGFβ1 and soluble PRELP Q155E using ELISA. The wells were coated with TGFβ1, and the bindings of soluble PRELP WT (blue) and Q155E (yellow) were measured. Bound PRELP was detected with anti-DDDDK-tag mAb HRP-DirecT, and the absorbance at 450 nm was measured after the TMB reaction.

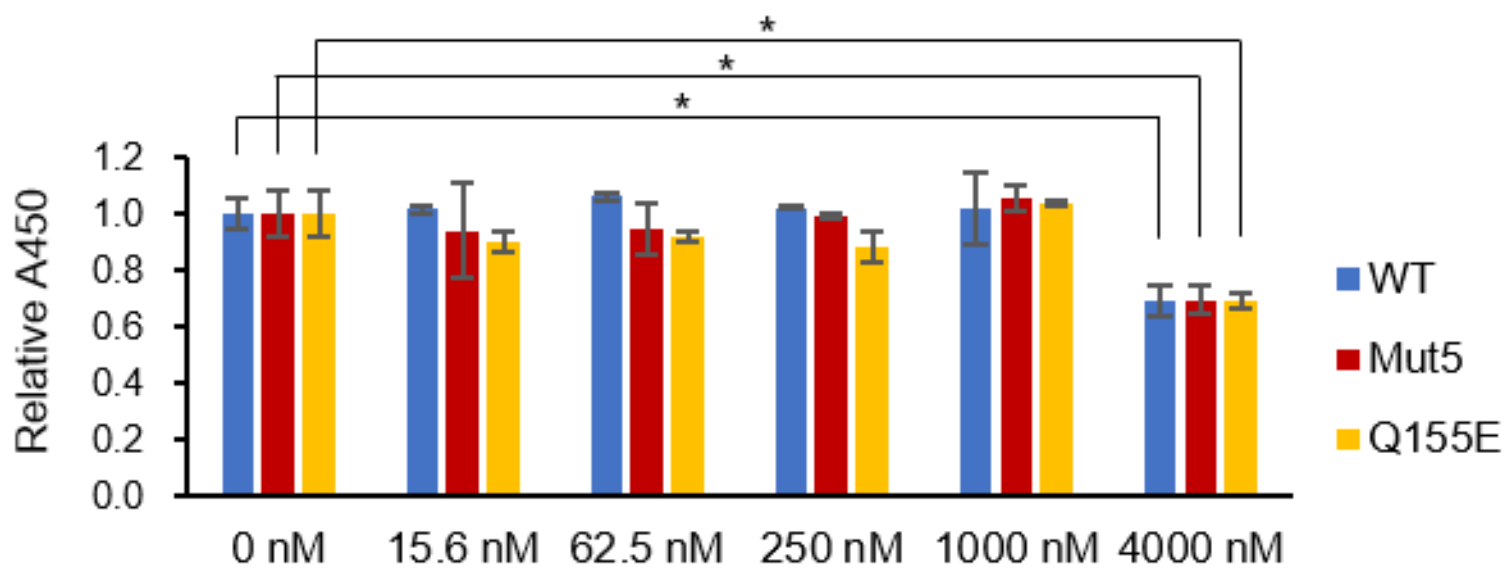

Figure S18. Influence of PRELP homodimerization on growth suppression of A549 cells. Viability of A549 cells treated with a range of concentrations of PRELP was evaluated using Cell Count Reagent SF. The average values of absorbance at 450 nm ± standard deviations are shown. ∗ indicates $p < 0.05$ between 0 nM and 4000 nM measurements.
